# Supplementary material for: Quantifying interactions in the active encounter complex of frustrated Lewis pairs
Source: Nat Commun. 2025 Apr 17;16:3666. doi: 10.1038/s41467-025-58965-2 (PMC12006534; doi:10.1038/s41467-025-58965-2)
Supplement: Supplementary file 1 — Supplementary Information [file 41467_2025_58965_MOESM1_ESM.pdf]

Supplementary Information

Quantifying Interactions in the Active Encounter Complex of Frustrated Lewis Pairs

Alastair T. Littlewood, Tao Liu, Laura. E. English, Linjiang Chen, Timothy A. Barendt, Andrew R. Jupp

Table of Contents

General....2
Spectrometer Details....2
Equipment Details....2
Chemical Suppliers....2
Chemical Synthesis....3
UV-vis of FLP systems in solution....5
Trimesitylphosphine and $B(C_6F_5)_3$....5
Alternative triarylphosphines with $B(C_6F_5)_3$....7
Titrations....9
Association Constant Determination of $P(mes)_3/B(C_6F_5)_3$ pair....9
Binding Data Analysis for $P(mes)_3/B(C_6F_5)_3$.....11
Titration of $P(dur)_3/B(C_6F_5)_3$ pair.....13
NMR Spectra....14
Trimesitylphosphine ($P(mes)_3$)....14
Tris(pentafluorophenyl)borane ($B(C_6F_5)_3$)....15
Tri(*o*-tolyl)phosphine (P(*o*-tol)$_3$)....16
Tris(2,5-dimethylphenyl)phosphine ($P(xyl)_3$)....17
Tris(2,3,5,6-tetramethylphenyl)phosphine ($P(dur)_3$)....18
Tris(pentamethylphenyl)phosphine) ($P(C_6Me_5)_3$)....19
Hydrogen Activation Experiments....21
Degradation Products....27
Computational Details....30
Binding configuration generation....30
$P(mes)_3/B(C_6F_5)_3$ binding energy landscape....31
$P(mes)_3/B(C_6F_5)_3$ binding energy landscape at the density functional theory (DFT) level....32
Time-dependent DFT (TD-DFT) calculations....33
Additional Figures....37
References....39

General

Spectrometer Details

All UV-vis data for the supramolecular titration experiments were collected and processed on a Biochrom Biowave 3 UV-visible Life Science Spectrophotometer (1) placed inside an MBraun ECO glovebox under an atmosphere of nitrogen, with oxygen levels maintained at < 0.1 ppm, and water levels at ≤ 0.6 ppm. The data were processed using Biochrom PVCViewer. For the time-dependent measurements, samples were prepared in the glovebox and placed in a cuvette fitted with an air-tight valve, and measured on an Agilent Technologies Cary 60 UV-vis Spectrophotometer (2) with an Agilent Technologies Single Cell Peltier (at 20°C). These data were processed using Cary WinUV Scan Application Version 5.1.3.1042. All data was subsequently transferred to Microsoft Excel Version 2203 and OriginPro 2025 for further processing and graphing. For NMR data, ^{1}H, ^{11}B, ^{19}F, and ^{31}P NMR spectra were recorded using either a Bruker AVIII400 (400MHz), or Bruker AV NEO 400 (400 MHz) spectrometer, both fitted with a BBFO "Smart" probe; all NMR data was processed on MestReNova x64-14.0.0-23239.

Equipment Details

All air-sensitive compounds were handled, prepared, and stored inside an MBraun ECO glovebox under an atmosphere of nitrogen, with oxygen levels maintained at < 0.1 ppm, and water levels at ≤ 0.6 ppm. The temperature inside the glovebox was measured as 301 K during the titrations. The balance used for measuring masses was a Nettler Toledo. Titration volumes were measured with 50 µL & 100 µL Hamilton syringes as well as 1 mL Fisher Scientific syringes with Braun needles, or Socorex Acura 826 micropippettes. For the time dependent measurements, a custom produced quartz cuvette (10 mm Light Path) with an attached Schlenk valve was utilised to allow measurements to be taken outside of the glovebox environment while maintaining an inert atmosphere. The cuvettes used for all remaining measurements were either a Hellma Analytics Semi-Micro Cell 114B-QS (10 mm Light Path) or Hellma Analytics Ultra-Micro Cell 105-201-15-40 (10 mm Light Path). The ampoule used in the hydrogen activation experiments was a custom-made long neck 15 mL Schlenk ampoule.

Chemical Suppliers

Trimesitylphosphine ($P(mes)_3$) was supplied by Alfa Aesar (98%) and tri(*o*-tolyl)phosphine was purchased from Thermo Fisher Scientific (98+%), both were used as received and stored in the glovebox at room temperature. Two sources of tris(pentafluorophenyl)borane ($B(C_6F_5)_3$) were used: all titration experiments with $P(mes)_3$ used $B(C_6F_5)_3$ supplied by TCI (> 98.0%), experiments with other triarylphosphines utilised synthesised $B(C_6F_5)_3$ (see Chemical Synthesis section for details), all were stored within the glovebox freezer at −35 °C. Magnesium turnings were supplied by Sigma Aldrich and flame dried under vacuum prior to use. PCl_3 was purchased from Sigma Aldrich and distilled prior to use. 1-Bromo-2,6-dimethylbenzene, 1-bromo-2,3,5,6-

tetramethylbenzene and bromopentamethylbenzene were all purchased from Thermo Scientific and used as received. Tetrahydrofuran (THF) and diethyl ether (Et_2O) were purchased from Fisher, dried over sodium/benzophenone and distilled before storage in a J. Young's ampoule over 3Å molecular sieves. Hexane was purchased from Sigma Aldrich, and $CDCl_3$ (1% v/v TMS) purchased from Sigma Aldrich; all were degassed and dried over 3Å molecular sieves and stored in J Young's ampoules. Toluene was supplied by the department SPS (solvent purification system), and was degassed and dried over a potassium mirror. d_8-toluene was purchased from Sigma Aldrich, dried over calcium hydride, degassed and stored in a J Young's ampoule over a potassium mirror. The 5% Hydrogen/Nitrogen gas (150318-L) was supplied by BOC and used as received.

Chemical Synthesis

Synthesis of tris(2,6-dimethylphenyl)phosphine)

A freshly prepared solution of 2,6-dimethylphenylmagnesium bromide in THF (49 mL, 0.91 M, 44.5 mmol, 3.2 eq.) was added dropwise, over 1 hour, to a solution of PCl_3 (1.2 mL, 1.98 g, 13.8 mmol, 1 eq.) in 40 mL THF at −78°C. The resulting solution was then allowed to warm to room temperature then stirred overnight. The solvent was removed *in vacuo* and the resulting solid extracted with 125 mL toluene to give a pale yellow solution. Removal of the solvent *in vacuo* produced an off-white solid which was recrystallised from hexane. Yield: 3.30 g, 69%. ^{1}H NMR (400 MHz, d_8-toluene) δ 6.99-6.95 (m, 3H, Ar-C*H*), 6.85-6.82 (m, 6H, Ar-C*H*), 2.15 (s, 18H, CH_3). ^{31}P{^{1}H} NMR (162 MHz, d_8-toluene) δ −34.6. NMR data are consistent with literature values.[1]

Synthesis of tris(2,3,5,6-tetramethylphenyl)phosphine)

A freshly prepared solution of 2,3,5,6-tetramethylphenyl magnesium bromide in THF (39 mL, 0.77 M, 30.0 mmol, 3.3 eq.) was added dropwise, over 1 hour, to a solution of PCl_3 (1 mL, 1.25 g, 9.1 mmol, 1 eq.) in 10 mL THF at −78°C. The resulting solution was then allowed to warm to room temperature then stirred overnight. The solvent was removed *in vacuo* and the resulting solid extracted with 100 mL toluene to give a pale yellow solution. Removal of the solvent *in vacuo* produced an off-white solid which was recrystallised from hexane. Yield: 2.00 g, 51%. ^{1}H NMR (400 MHz, d_8-toluene) δ 6.88 (s, 3H, Ar-C*H*), 2.23 (s, 18H, CH_3), 2.07 (s, 18H, CH_3). ^{31}P{^{1}H} NMR (162 MHz, d_8-toluene) δ −29.1. NMR data are consistent with literature values.[2, 3]

Synthesis of tris(pentamethylphenyl)phosphine)

A freshly prepared solution of pentamethylphenylmagnesium bromide in THF (59 mL, 0.25 M, 15 mmol, 3.3 eq.) was added dropwise, over 1 hour, to a solution of PCl_3 (0.4 mL, 0.62 g, 4.5 mmol, 1 eq.) in 20 mL THF at −78°C. The resulting solution was then allowed to warm to room temperature then stirred overnight. The solvent was removed *in vacuo* and the resulting solid extracted with 150 mL hexane to give a pale yellow solution. Removal of the solvent *in vacuo* produced an off-white solid which was recrystallised from hexane. Yield: 0.66 g, 23%. ^{1}H NMR (300 MHz, $CDCl_3$) δ 2.25 (s, 9H, CH_3), 2.16 (s, 18H, CH_3), 2.07 (s, 18H, CH_3). $^{31}P\{^{1}H\}$ NMR (121 MHz, $CDCl_3$) δ −25.1. NMR data are consistent with literature values.[4]

Synthesis of tris(pentafluorophenyl)borane ($B(C_6F_5)_3$)

A freshly prepared solution of pentafluorophenylmagnesium bromide in Et_2O (200 mL, 0.6 M, 120 mmol, 3 eq.) was added dropwise to a vigorously stirred solution of $BF_3{\cdot}Et_2O$ (5 mL, 5.75 g, 40 mmol, 1 eq.) in 80 mL toluene at 0°C. The reaction was then allowed to warm to room temperature and the Et_2O removed *in vacuo*. The resulting toluene solution was then heated to 98°C for 1 hour using a water bath before cooling to room temperature and removing the remaining solvent *in vacuo*. The product was extracted 3 times with warm hexane and crystallised by cooling the hexane solutions to −30°C. Yield: 15.302 g, 65%. Analytically pure material is obtained by two consecutive sublimations under dynamic vacuum (1×10^{-2} mbar) at 90°C. Average sublimation yield: 85%. $^{11}B\{^{1}H\}$ NMR (128 MHz, $CDCl_3$) δ 57.8 (br, s). $^{19}F\{^{1}H\}$ NMR (376 MHz, $CDCl_3$) δ −128.0 (s, 6F), −143.0 (s, 3F), −159.9 (m, 6F). NMR data are consistent with literature values.[5]

UV-vis of FLP systems in solution

Trimesitylphosphine and $B(C_6F_5)_3$

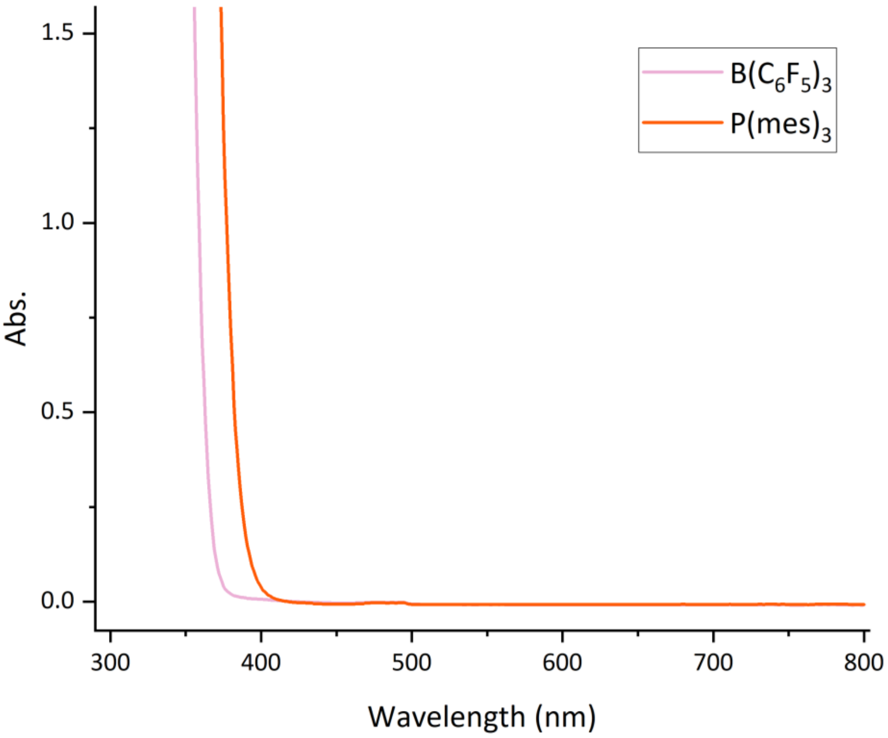

Fig. S1: Lewis acid & base separately dissolved in solution (10 mM), showing an absence of the charge transfer absorption band. Spectrophotometer 2.

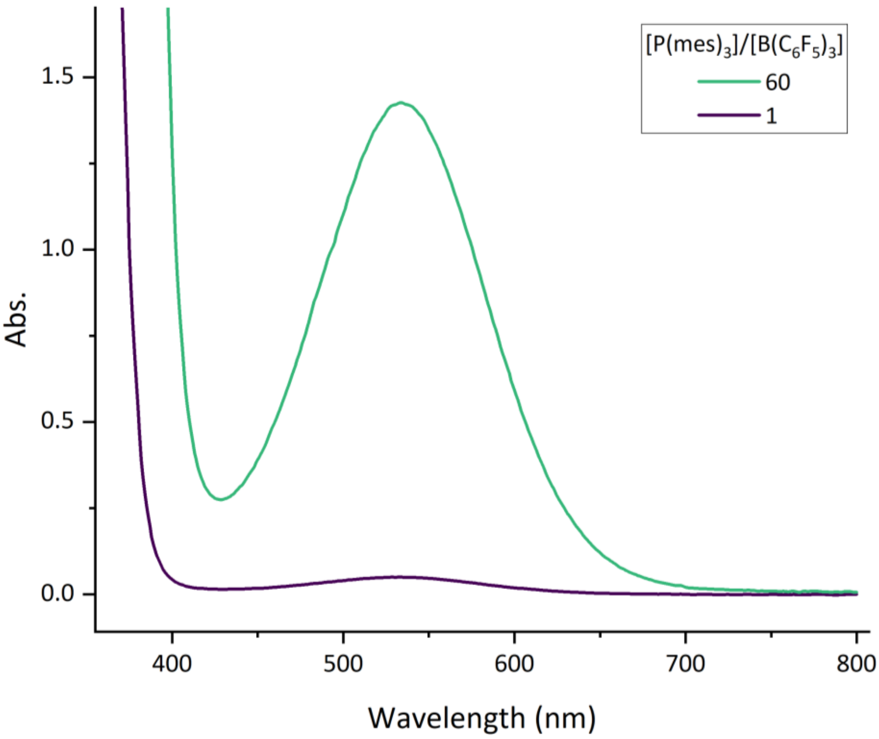

Fig. S2: Comparison of absorption bands for 1:1 (5 mM) vs. 1:60 ($B(C_6F_5)_3$ at 5 mM, $P(mes)_3$ at 300 mM) ratios in toluene. Spectrophotometer 1.

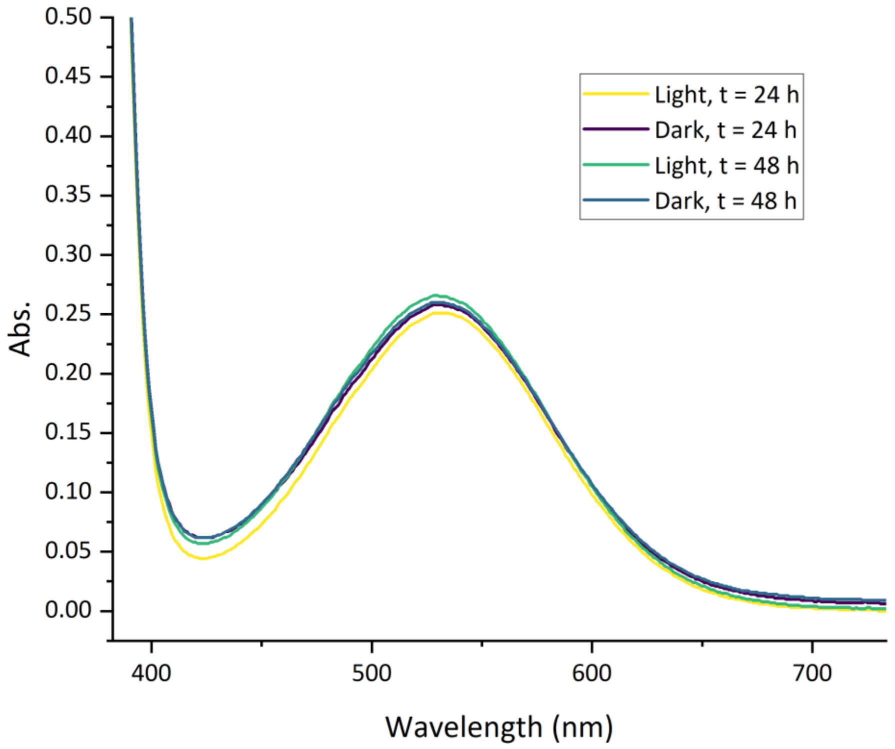

Fig. S3: Comparison of absorption bands for a 1:1 $P(mes)_3$:$B(C_6F_5)_3$ (25 mM) with one sample wrapped in foil between scans, and one left exposed to ambient lighting conditions, λ_{max} remaining consistent throughout. Samples left in glovebox during experiment. Spectrophotometer 1.

Alternative triarylphosphines with $B(C_6F_5)_3$

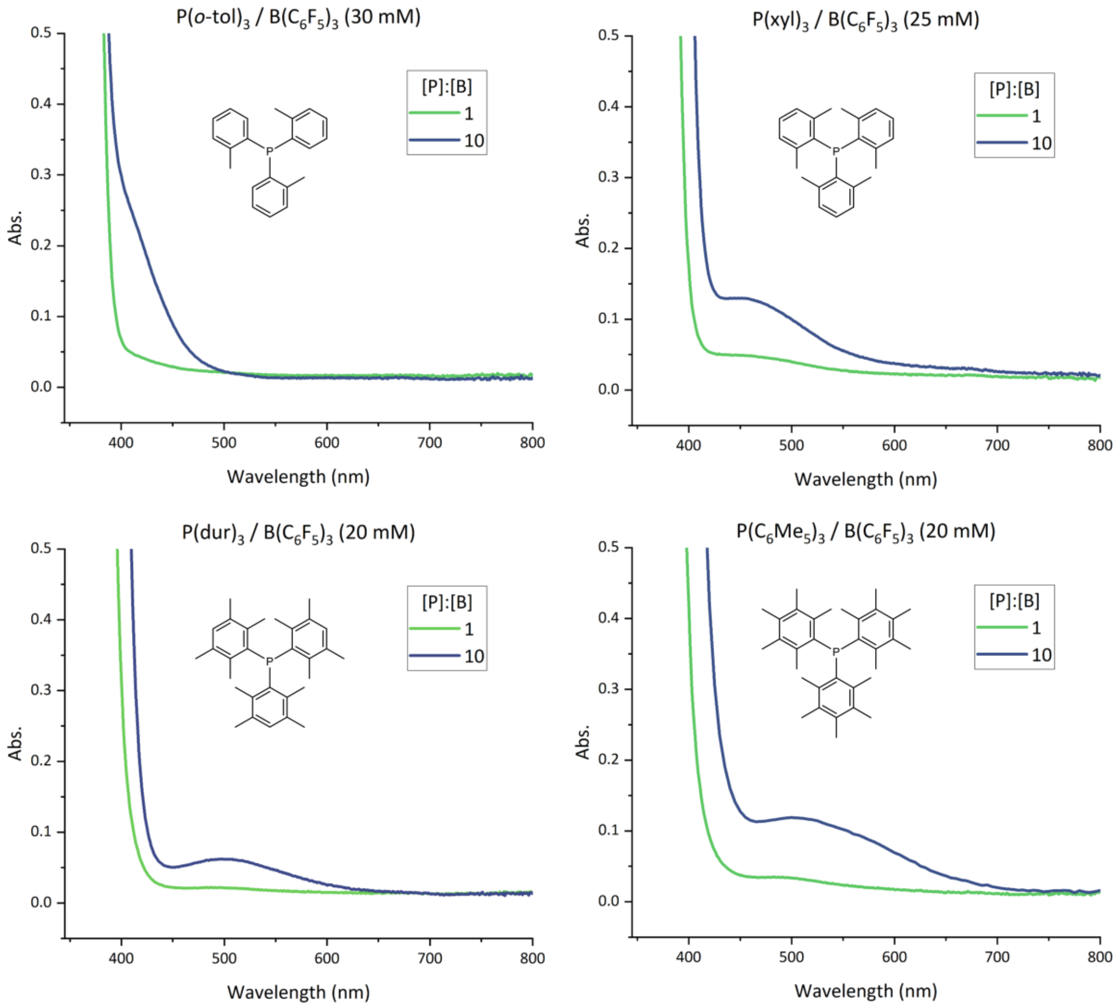

Fig. S4: Comparison of charge-transfer bands of various triarylphosphines with $B(C_6F_5)_3$ in toluene; the concentration of $B(C_6F_5)_3$ shown in brackets was determined by the maximum solubility of the phosphine in toluene for the 10:1 ratio. The λ_{max} for each phosphine with $B(C_6F_5)_3$ at 20 °C was: P(*o*-tolyl)$_3$: N/A, P(xyl)$_3$: 451 nm, P(dur)$_3$: 496 nm, $P(C_6Me_5)_3$: 500 nm. Spectrophotometer 2.

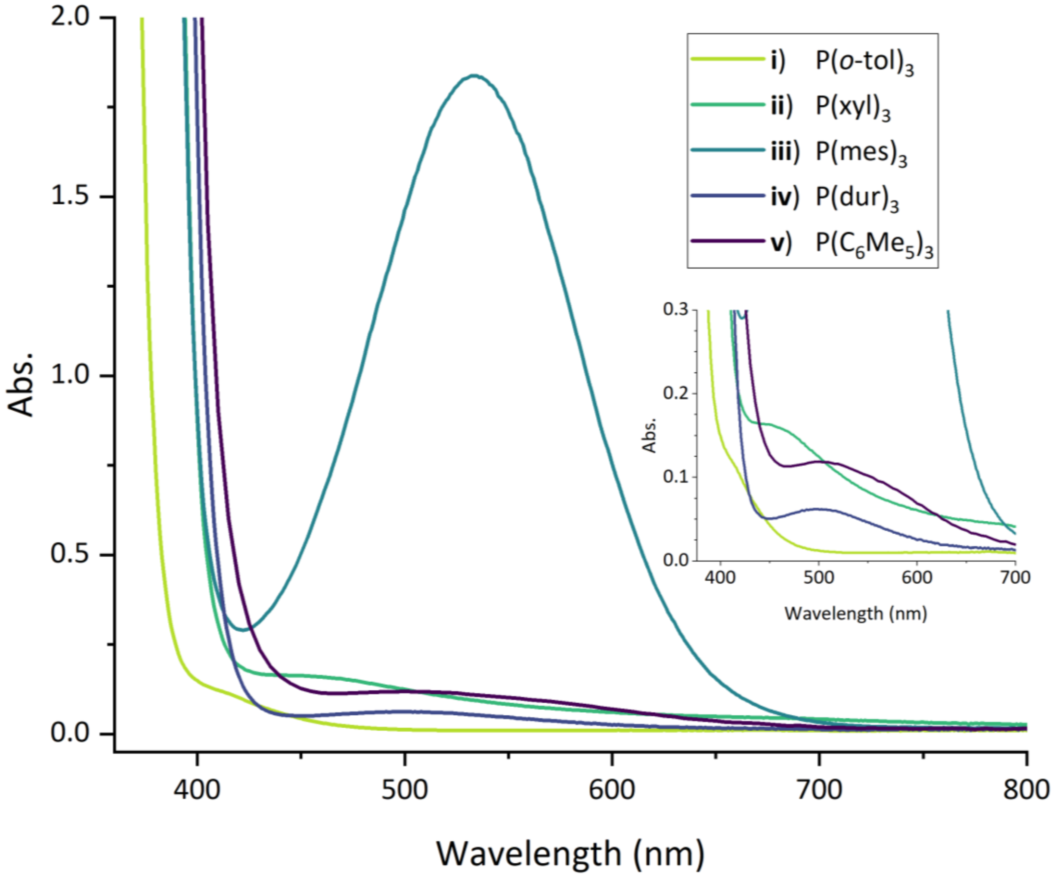

Fig. S5: Stacked quantitative comparison of charge-transfer bands of various triarylphosphines in a 10:1 ratio of [P]:[B], with $B(C_6F_5)_3$ maintained at 20 mM in toluene at 20 °C. Spectrophotometer 2.

Titrations

Association Constant Determination of $P(mes)_3/B(C_6F_5)_3$ pair.

For the association constant (K_a) determination, stock solutions of 380 mM $P(mes)_3$ and 100 mM $B(C_6F_5)_3$ were made respectively using the same source of dried and degassed toluene in a nitrogen glovebox. For the samples, screw top glass vials were heated to 85 °C for at least 30 minutes before being taken into the glovebox. Each vial was labelled, and a combination of syringes (as outlined in the equipment details) were used to measure out the correct volumes of each stock solution and any additional solvent that was required, producing the correct resultant concentrations (5 mM $B(C_6F_5)_3$) for the desired ratios from 1:1 to 60:1 ($[P(mes)_3]:[B(C_6F_5)_3]$) at 1 mL total volume per sample. Each vial had a screw top lid applied and sealed further with electrical tape to minimise contamination or evaporation within the glovebox. For the data collection, each sample was removed from its vial and analysed within the glovebox on a Biochrom spectrometer (1) as outlined in the equipment section. The data was plotted on OriginLab and analysed as described in the next section.

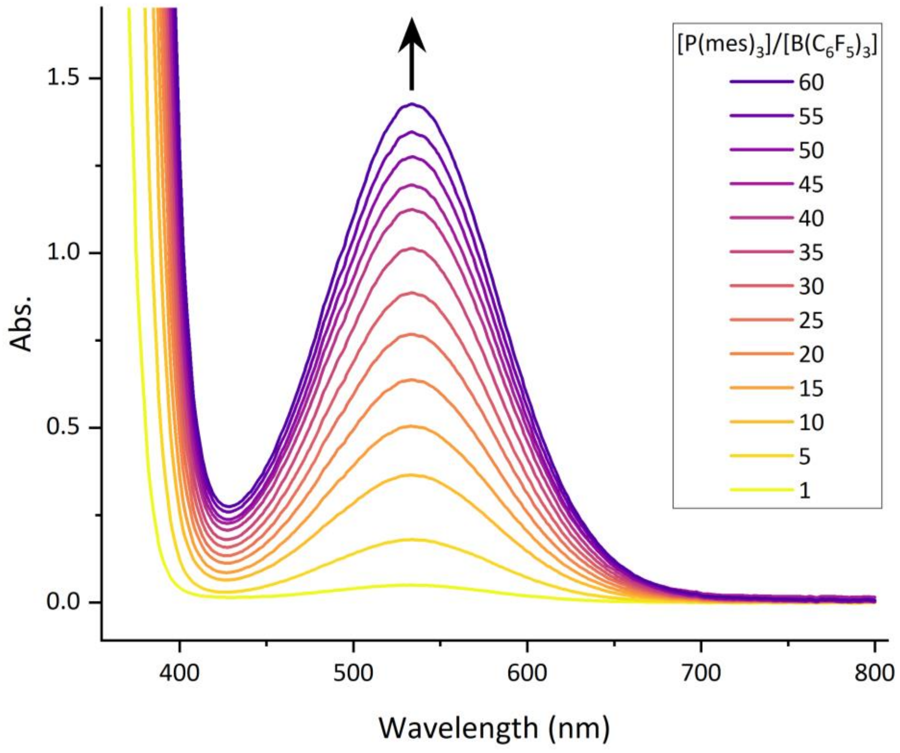

Fig. S6: Stacked overlay of all ratios of $P(mes)_3$ to $B(C_6F_5)_3$ in toluene. Spectrophotometer 1.

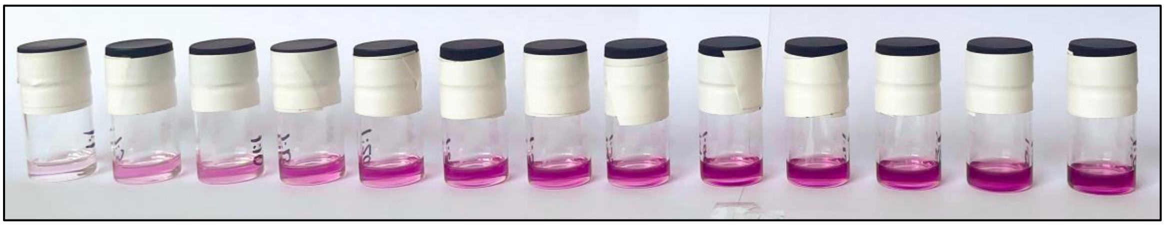

Fig. S7: Photograph of FLP sample vials from 1:1 to 60:1 of $P(mes)_3/B(C_6F_5)_3$, with $B(C_6F_5)_3$ maintained at 5 mM in toluene.

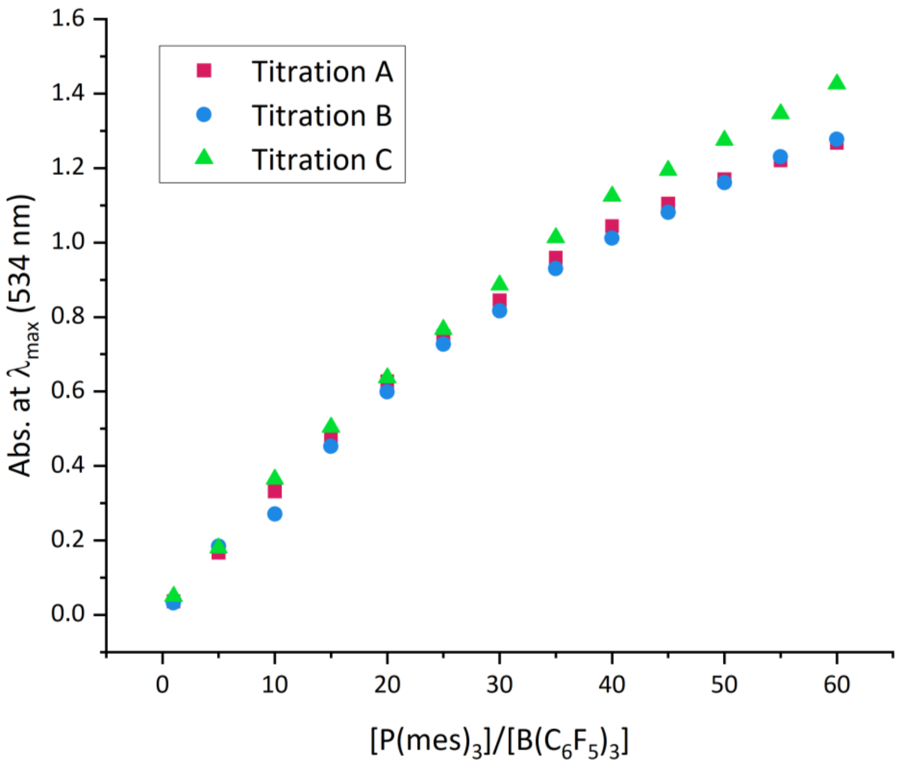

Fig. S8: Absorbance vs. $[P(mes)_3]/[B(C_6F_5)_3]$ ratio of all titrations in toluene ($B(C_6F_5)_3$ concentration maintained at 5 mM). Spectrophotometer 1.

Ratio ($[P(mes)_3]$:$[B(C_6F_5)_3]$)	Absorbance at λ_{max} (534 nm)		
	Titration A	Titration B	Titration C
1:1	0.036	0.032	0.050
5:1	0.167	0.184	0.180
10:1	0.331	0.271	0.365
15:1	0.481	0.453	0.504
20:1	0.627	0.599	0.637
25:1	0.749	0.727	0.767
30:1	0.845	0.816	0.886
35:1	0.959	0.930	1.013
40:1	1.044	1.012	1.125
45:1	1.105	1.081	1.194
50:1	1.170	1.161	1.275
55:1	1.221	1.230	1.346
60:1	1.268	1.277	1.426

Table S1. Raw λ_{max} absorbance data for each titration in toluene solvent.

Binding Data Analysis for $P(mes)_3/B(C_6F_5)_3$.

The UV-vis spectroscopic titration curves for $B(C_6F_5)_3$ (host) upon titration with up to 60 equivalents of $P(mes)_3$ (guest) in toluene are shown below. The association constant (K_a) was determined by multiwavelength, non-linear curve fitting using BindFit.[6, 7] The spectral region λ = 524 nm – 544 nm was used because this is where the largest changes in the charge transfer absorption band occurs and this region has no other conflicting absorbances (see Fig. S1 & S6). The best fit was obtained using a 1:1 host–guest stoichiometric model, with all fitting errors $\leq$ 0.4 %. Further justification for the 1:1 stoichiometric binding model comes from the random distribution of residuals (see Fig. S9) because deviation from the theoretical binding isotherm is caused by random experimental errors.[8] An average K_a (2.52 M^{-1}) was obtained from three titration experiments (A–C), with the relative standard deviation being 17.1%. The solid points (black squares) are experimental data and red lines are the theoretical binding isotherm. Alternative 1:2 and 2:1 host–guest stoichiometric binding models were ruled out due to non-sensical K_a values (e.g. negative K_a), and high fitting errors.

Titration A

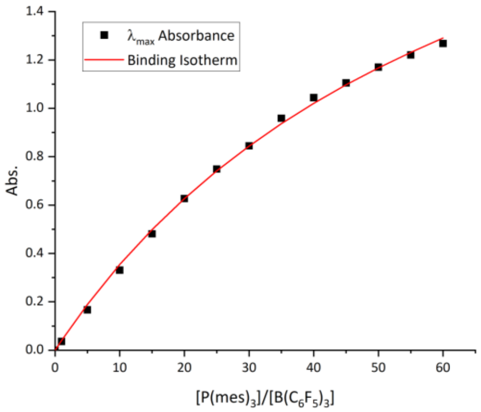

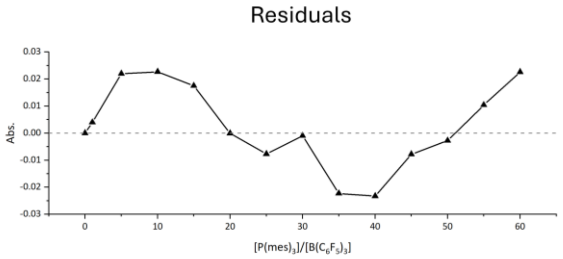

K_a = 3.02 M^{-1}

Fitting Error = ± 0.4 %

Titration B

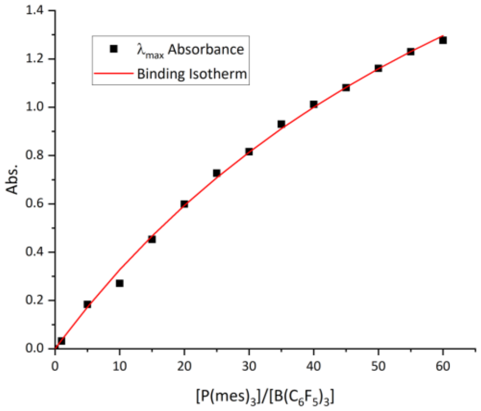

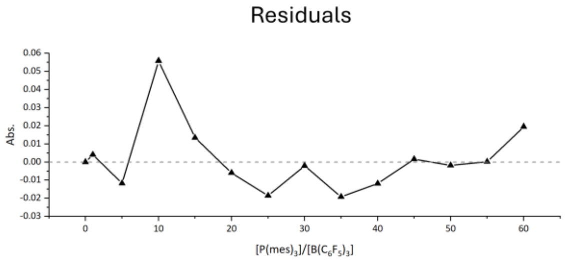

K_a = 2.23 M^{-1}

Fitting Error = ± 0.4 %

Titration C

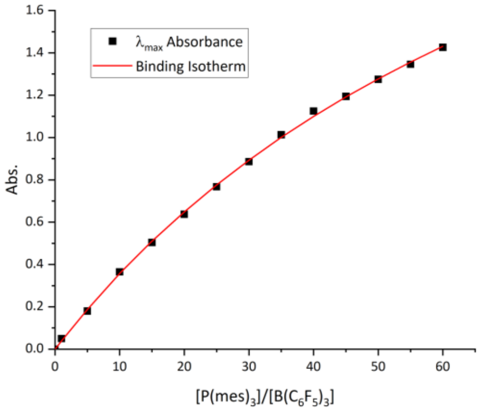

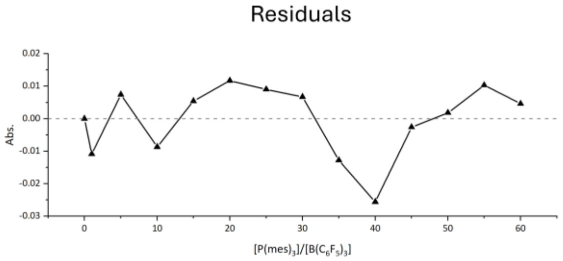

K_a = 2.32 M^{-1}

Fitting Error = ± 0.2 %

Fig. S9: Binding isotherms of all $P(mes)_3/B(C_6F_5)_3$ titrations (A–C) in toluene with their respective K_a, fitting error, and residuals ($B(C_6F_5)_3$ concentration maintained at 5 mM for each titration).

Titration of $P(dur)_3/B(C_6F_5)_3$ pair.

Stock solutions of 250 mM $P(dur)_3$ and 500 mM $B(C_6F_5)_3$ were made respectively using the same source of dried and degassed toluene. For the samples, screw top glass vials were heated to 85 °C for at least 30 minutes before being taken into the glovebox. Each vial was labelled, and micropipettes (as outlined in the equipment details) were used to measure out the correct volumes of each stock solution and any additional solvent that was required, producing the correct resultant concentrations (20 mM $B(C_6F_5)_3$) for the desired ratios from 1:1 to 22:1 ([$P(dur)_3$]:[$B(C_6F_5)_3$]) at 200 µL total volume per sample. Each vial had a screw top lid applied and sealed further with electrical tape to minimise contamination or evaporation within the glovebox. Given the lower absorbance levels for this system, small 100 µL cuvettes were used with Suba-Seal® septa and electrical tape to permit analysis on the more sensitive Cary-60 (Spectrophotometer 2), each sample being scanned immediately when removed from the glovebox. Prior to analysis using this method, samples of $P(dur)_3/B(C_6F_5)_3$ were made up, and the CT absorbance was found to be consistent for at least 5 minutes after the samples were removed from the glovebox using this method.

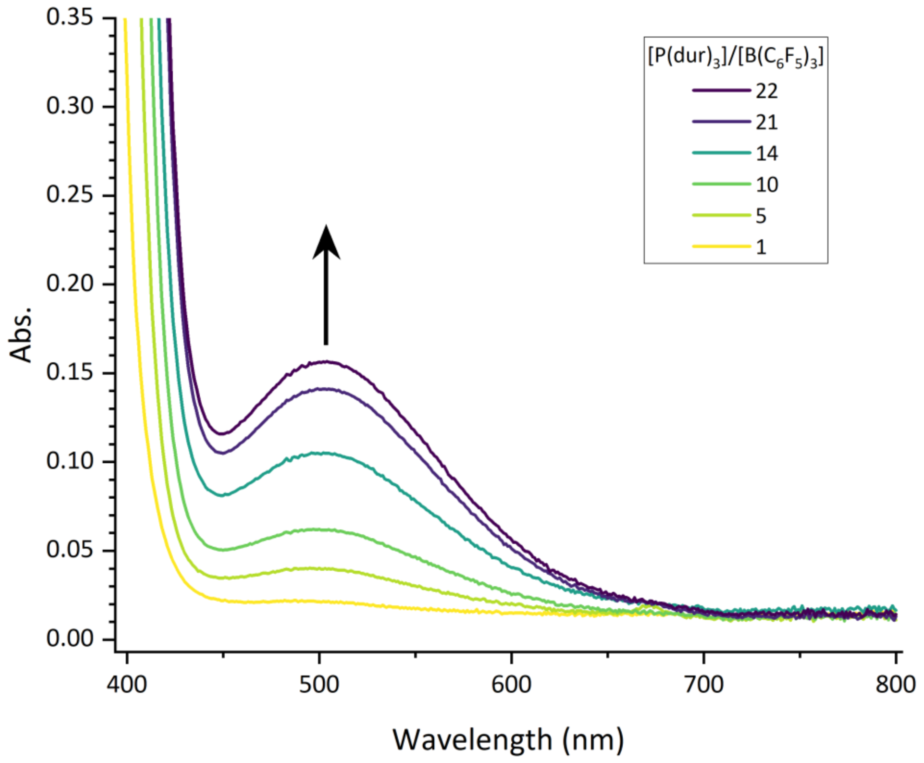

Fig. S10: Stacked overlay of $P(dur)_3$ to $B(C_6F_5)_3$ in toluene. Spectrophotometer 2.

NMR Spectra

Trimesitylphosphine (P(mes)$_3$)

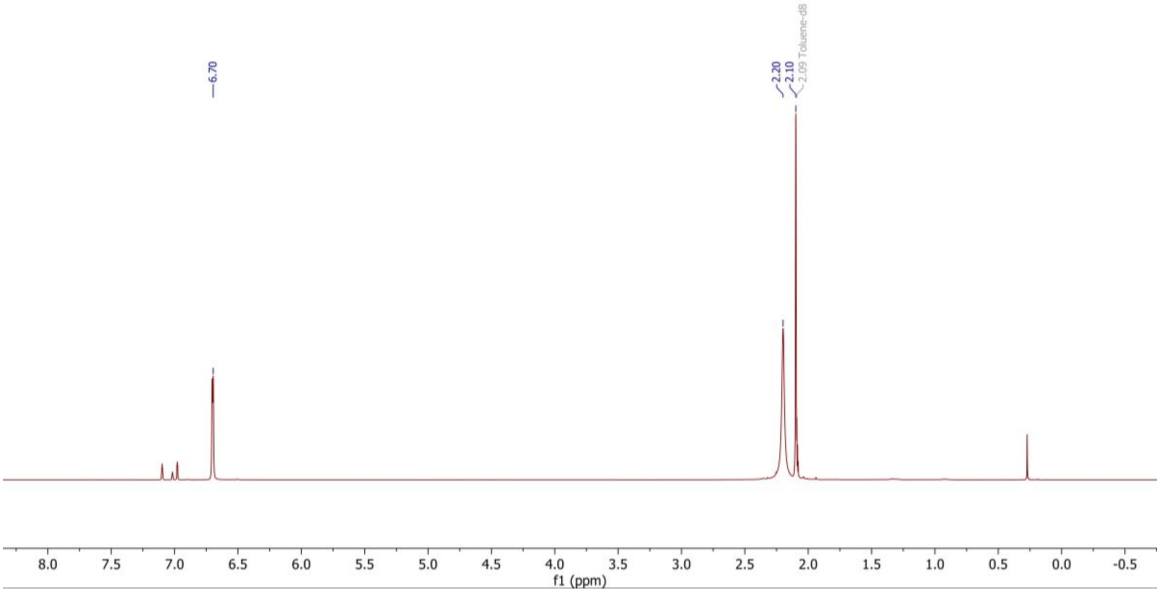

Fig. S11: ^{1}H NMR spectrum of P(mes)$_3$ (400 MHz, d_8-toluene) δ 6.70 (m, 2H, *m*-H), δ 2.20 (s, broad, 6H, *o*-CH$_3$), δ 2.10 (s, 3H, *p*-CH$_3$) (under N$_2$ atmosphere). Residual silicone grease observed at 0.26 ppm.

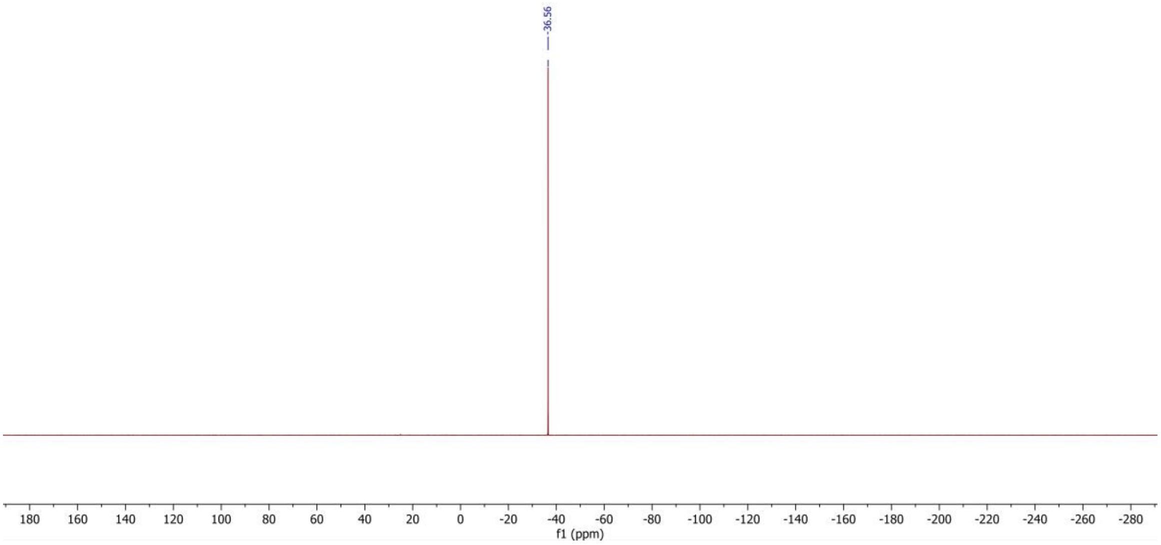

Fig. S12: ^{31}P{^{1}H} NMR spectrum of P(mes)$_3$ (162 MHz, d_8-toluene) δ −36.56 (under N$_2$ atmosphere).

Tris(pentafluorophenyl)borane ($B(C_6F_5)_3$)

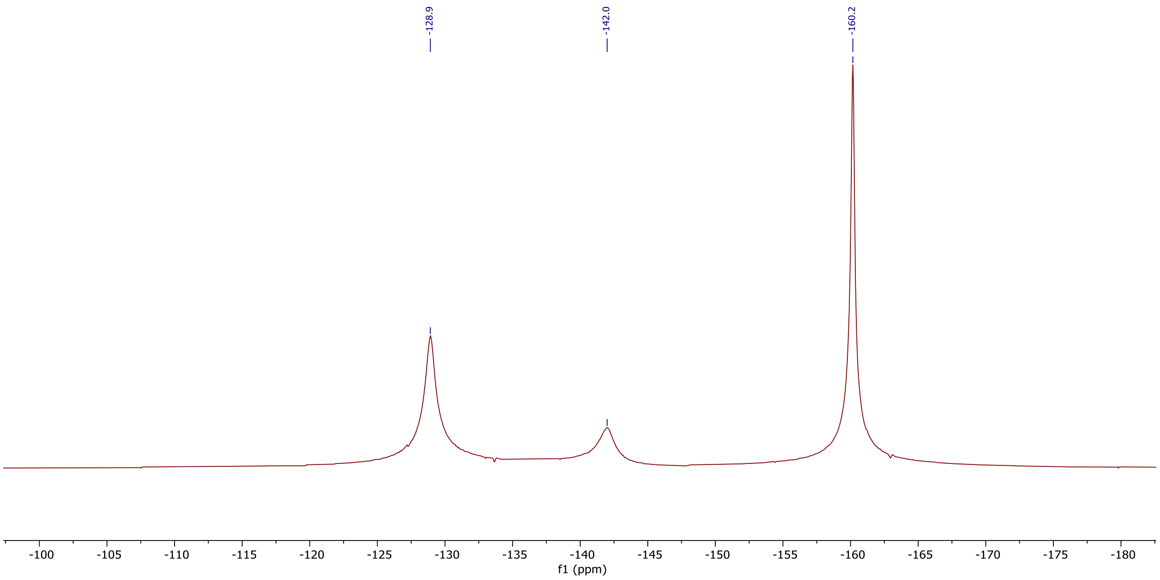

Fig. S13: ^{19}F NMR spectrum of $B(C_6F_5)_3$ (376 MHz, toluene) δ −128.9 (s, 6F, *o*-F), −142.0 (s, 3F, *p*-F), −160.2 (s, 6F, *m*-F), (under N_2 atmosphere).

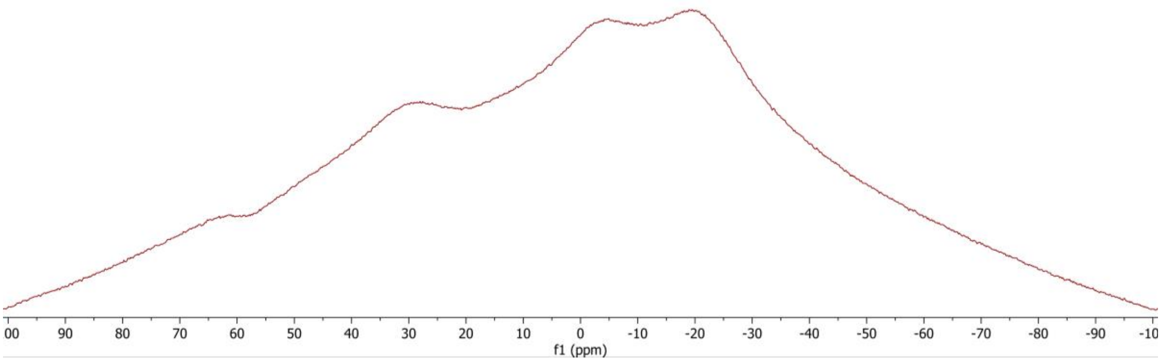

Fig. S14: ^{11}B NMR spectrum of $B(C_6F_5)_3$ (128 MHz, toluene) δ 61.9 (*c.f.* background signal in Fig. S15).

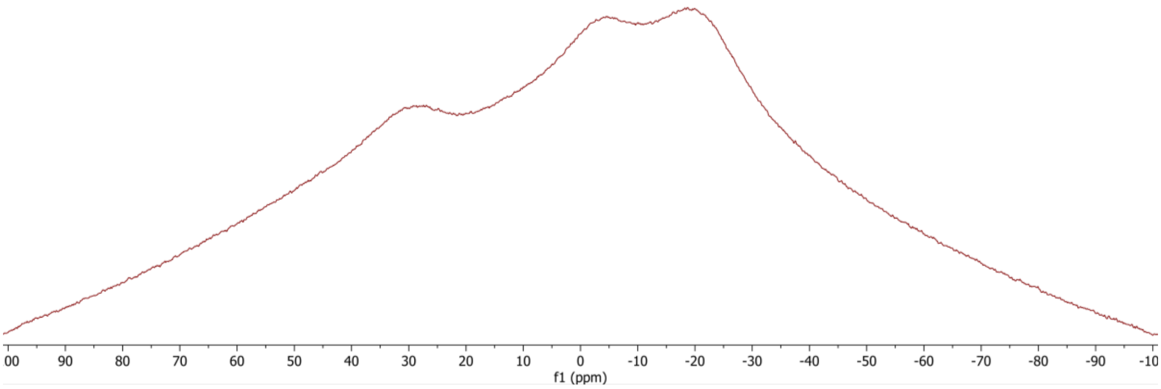

Fig. S15: ^{11}B NMR spectrum of toluene solvent blank (128 MHz, toluene) showing background signal from borosilicate glass.

Tri(*o*-tolyl)phosphine (P(*o*-tol)$_3$)

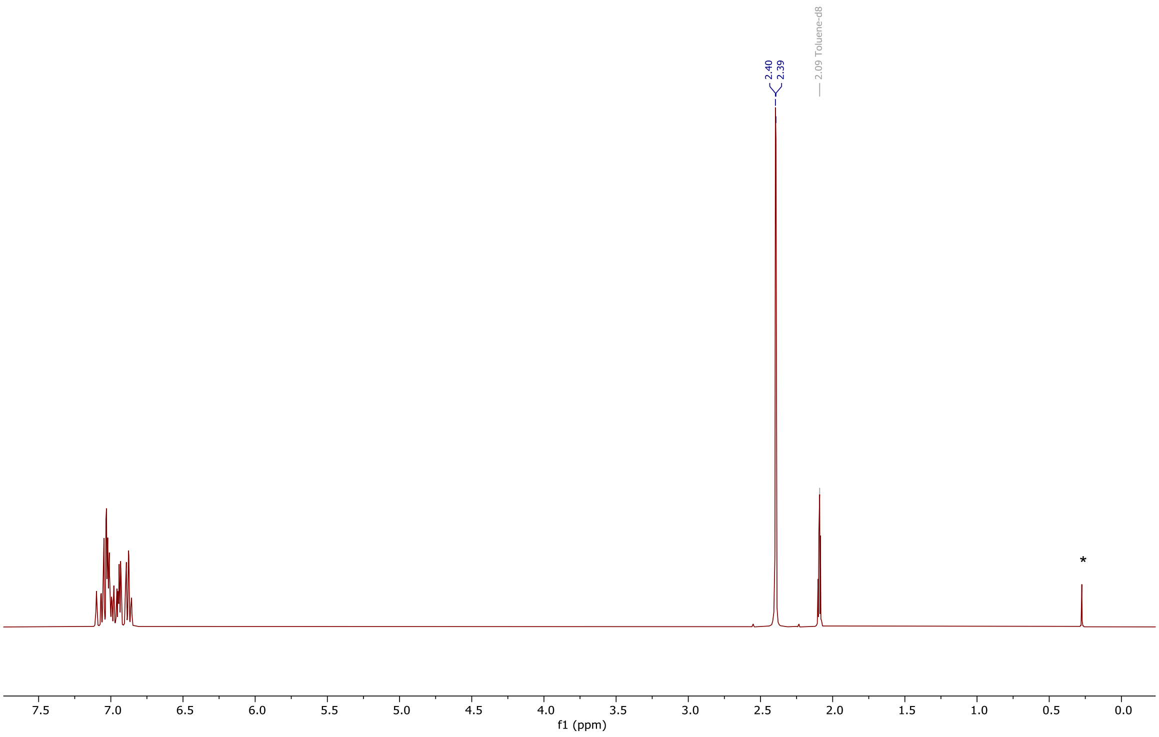

Fig. S16: ^{1}H NMR of tris(*o*-tolyl)phosphine) (400 MHz, d_8-toluene), *=silicon grease.

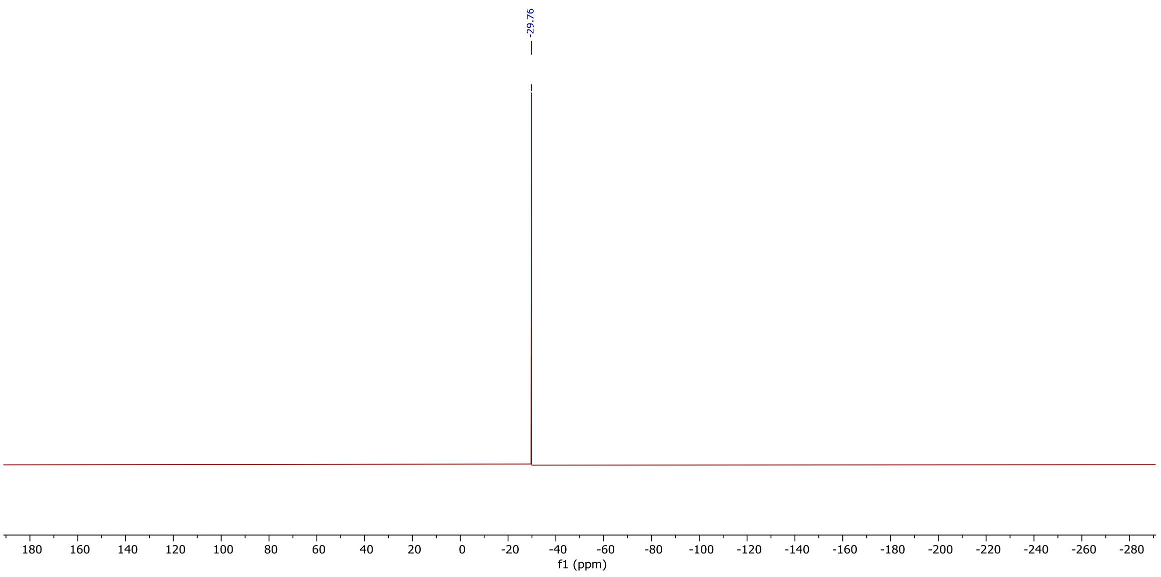

Fig. S17: $^{31}P\{^{1}H\}$ NMR of tris(*o*-tolyl)phosphine) (162 MHz, d_8-toluene).

Tris(2,5-dimethylphenyl)phosphine (P(xyl)$_3$)

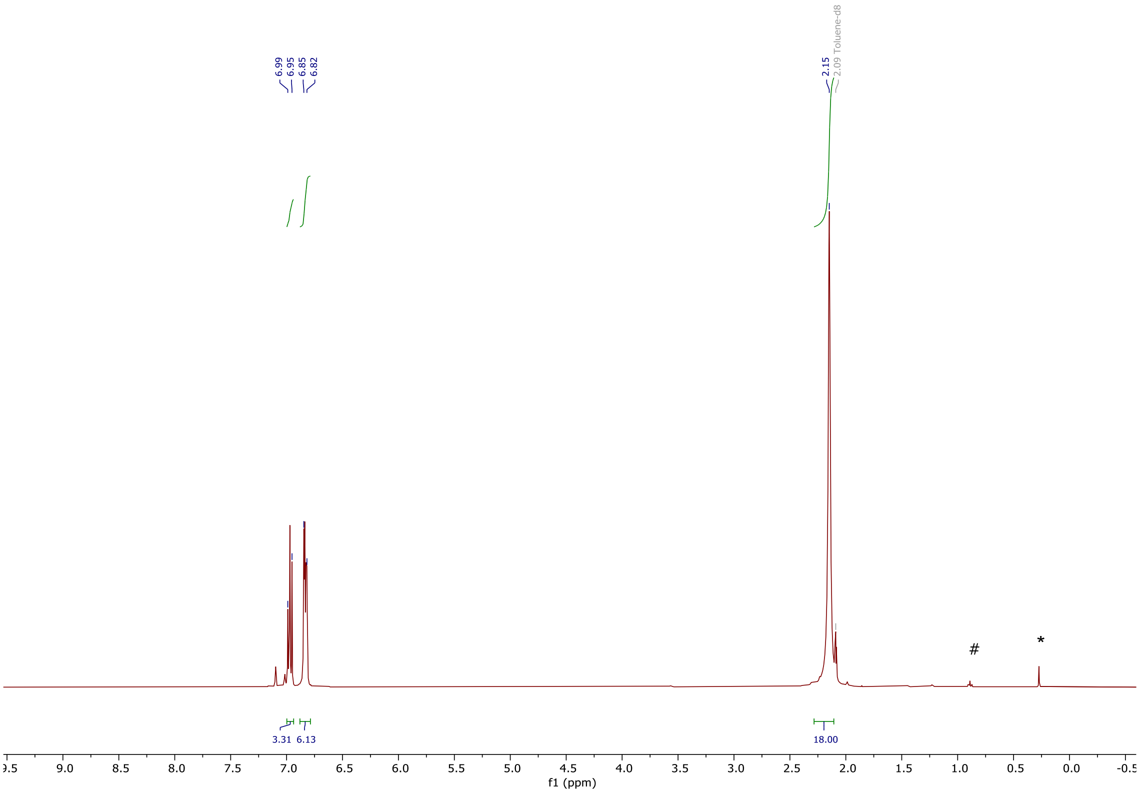

Fig. S18: ^{1}H NMR of tris(2,6-dimethylphenyl)phosphine) (400 MHz, d_8-toluene), *=silicon grease, #=residual hexane.

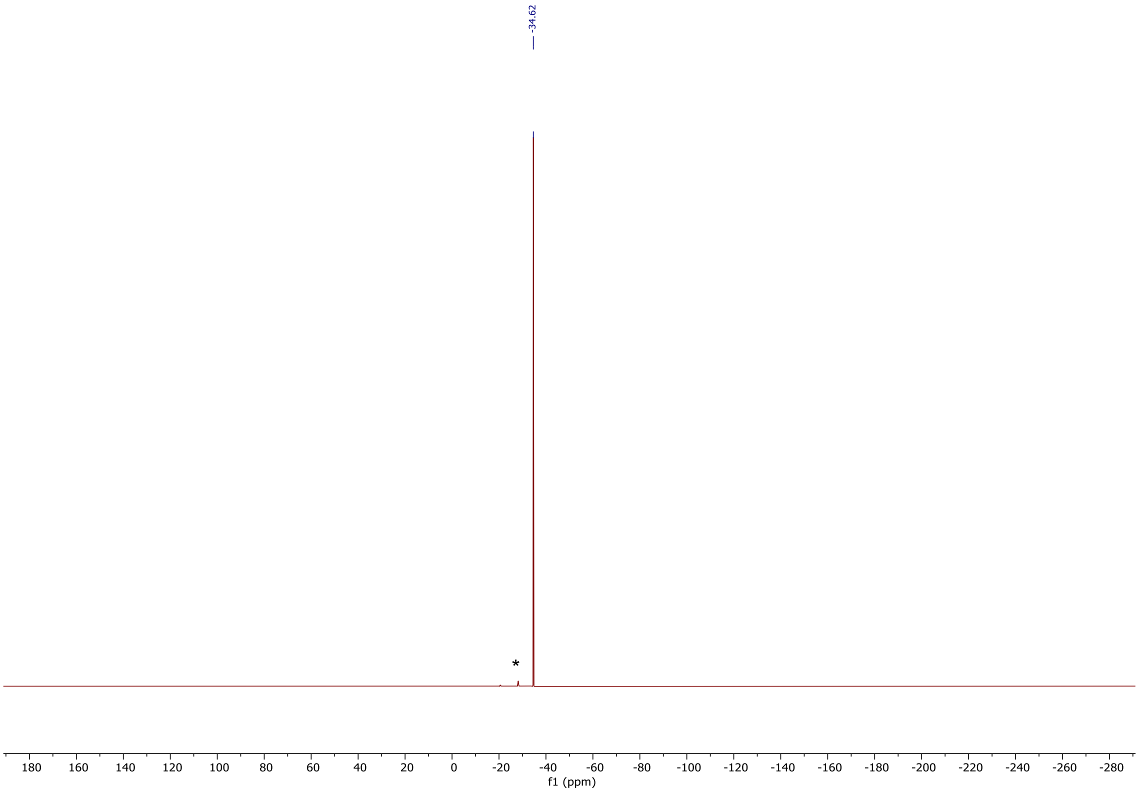

Fig. S19: $^{31}P\{^1H\}$ NMR of tris(2,6-dimethylphenyl)phosphine) (162 MHz, d_8-toluene), *= P_2Ar_4.

Tris(2,3,5,6-tetramethylphenyl)phosphine (P(dur)$_3$)

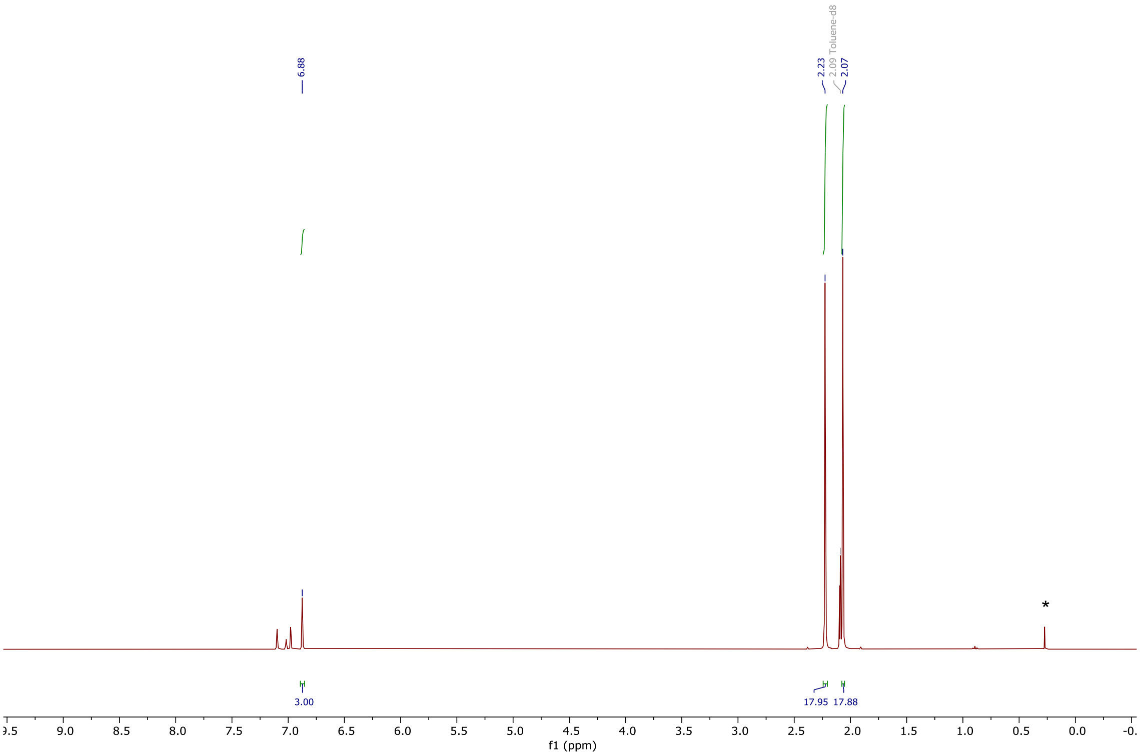

Fig. S20: ^1H NMR of tris(2,3,5,6-tetramethylphenyl)phosphine) (400 MHz, d_8-toluene), *=silicon grease.

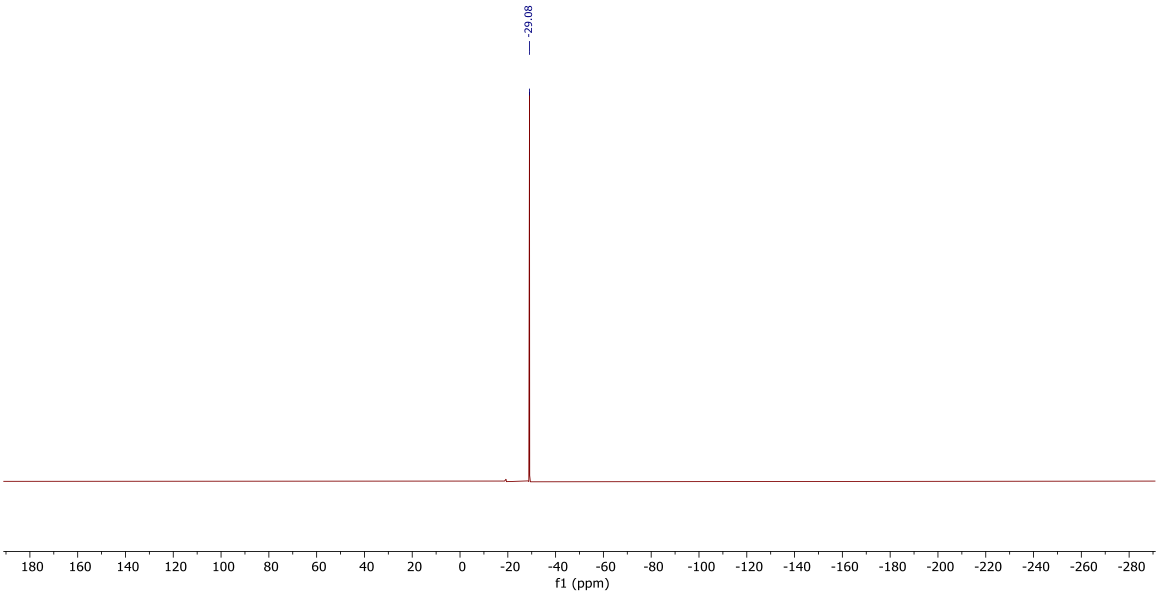

Fig. S21: $^{31}P\{^{1}H\}$ NMR of tris(2,3,5,6-tetramethylphenyl)phosphine) (162 MHz, d_8-toluene).

Tris(pentamethylphenyl)phosphine) ($P(C_6Me_5)_3$)

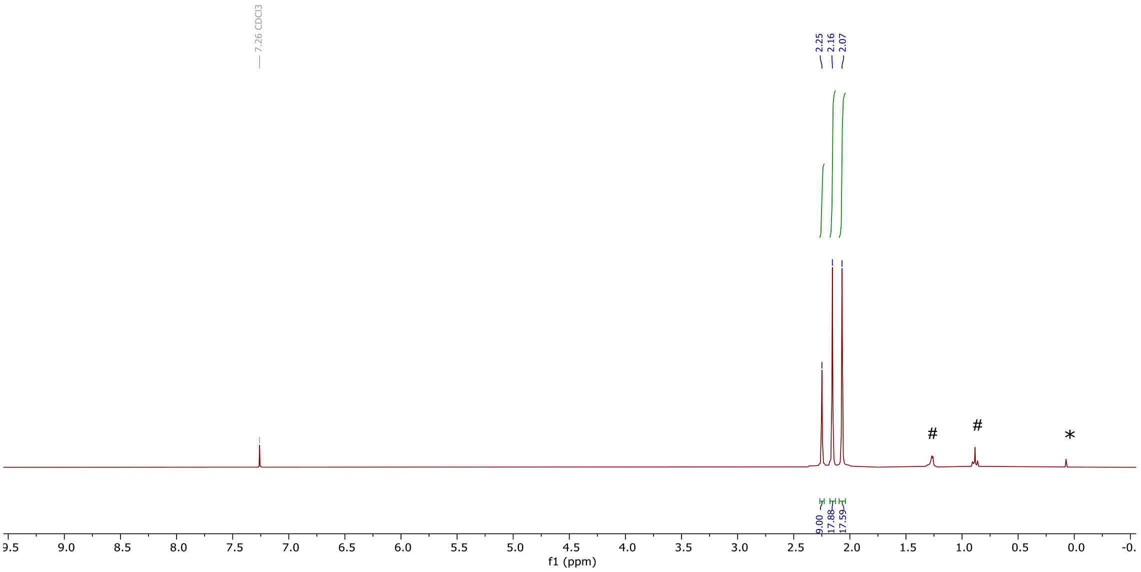

Fig. S22: ^{1}H NMR of tris(pentamethylphenyl)phosphine) (400 MHz, $CDCl_3$), *=silicon grease, #=residual hexane.

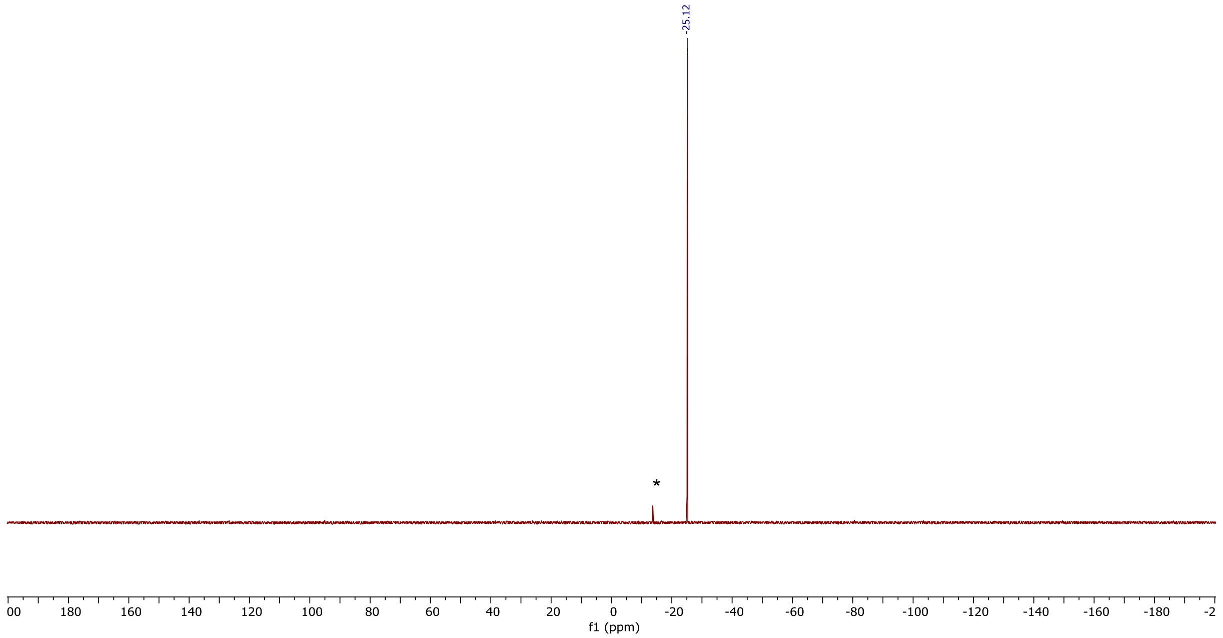

Fig. S23: $^{31}P\{^{1}H\}$ NMR of tris(pentamethylphenyl)phosphine) (162 MHz, $CDCl_3$), *=P_2Ar_4.

Hydrogen Activation Experiments

Two FLP ratios, 1:1 and 10:1 ($P(mes)_3/B(C_6F_5)_3$) were formed in 5 mL of toluene ($B(C_6F_5)_3$ maintained at 5 mM) within a 15 mL ampoule before being positioned in the same orientation within a Cary-60 spectrophotometer placed inside a fumehood (with cuvette holder removed and replaced with a stirrer plate). The solutions were stirred at 300 rpm, with a flow of 5% H_2 in N_2 gas (approx. 0.1 bar on the regulator) and a relief needle attached to a bubbler to ensure gas flow was maintained throughout. A UV-vis absorbance measurement was taken every minute until the λ_{max} absorbance was quenched to a point the absorbance reading was no longer changing, approximately 200 minutes. Hydrogen activated products were identified by multinuclear NMR spectroscopy through comparison to previous literature.[9]

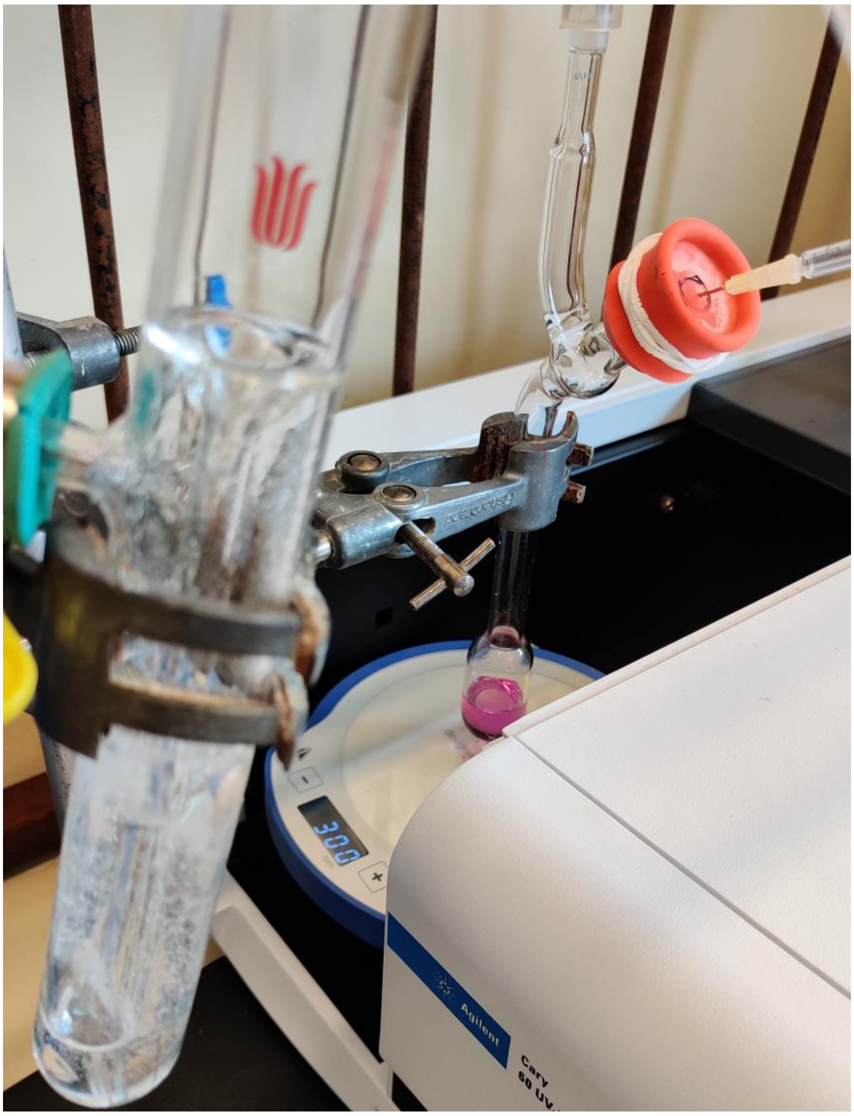

Fig. S24: Setup for hydrogen activation experiments. Ampoule containing $P(mes)_3/B(C_6F_5)_3$ in toluene is attached to a Schlenk line using 5% H_2 & 95% N_2 gas and suspended within Spectrophotometer 2. A relief needle is attached to an oil bubbler to observe gas flow throughout the system.

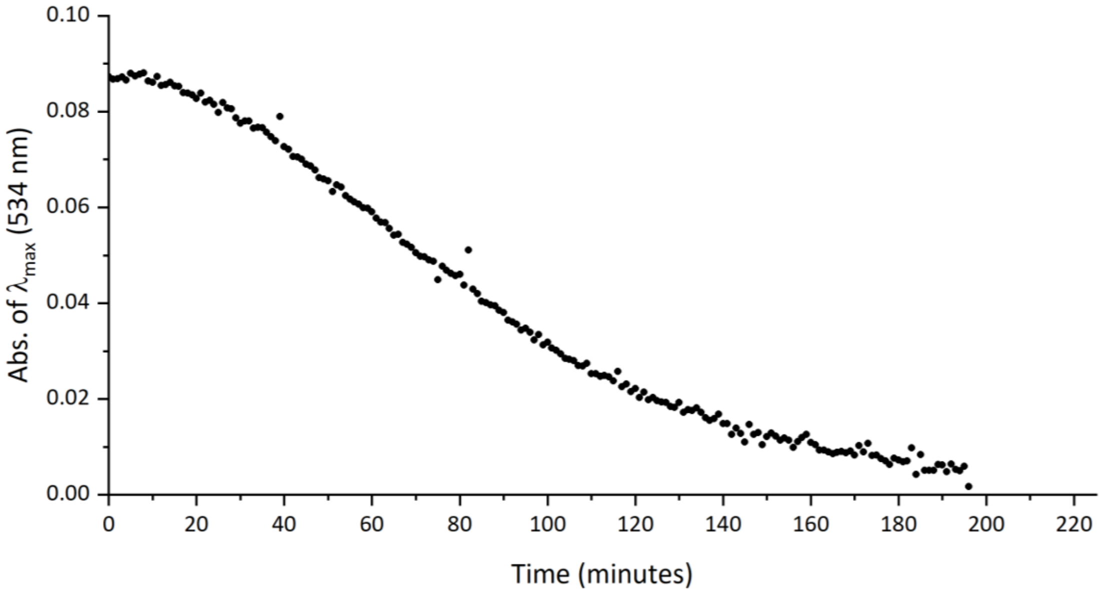

Fig. S25: Change in λ_{max} (534 nm) absorbance of a 1:1 P(mes)$_3$/B(C_6F_5)$_3$ FLP sample (5 mM in toluene) over time under a flow of 5% H_2 & 95% N_2 gas set at 0.1 bar. Half-life ($t_{½}$) = 88 minutes.

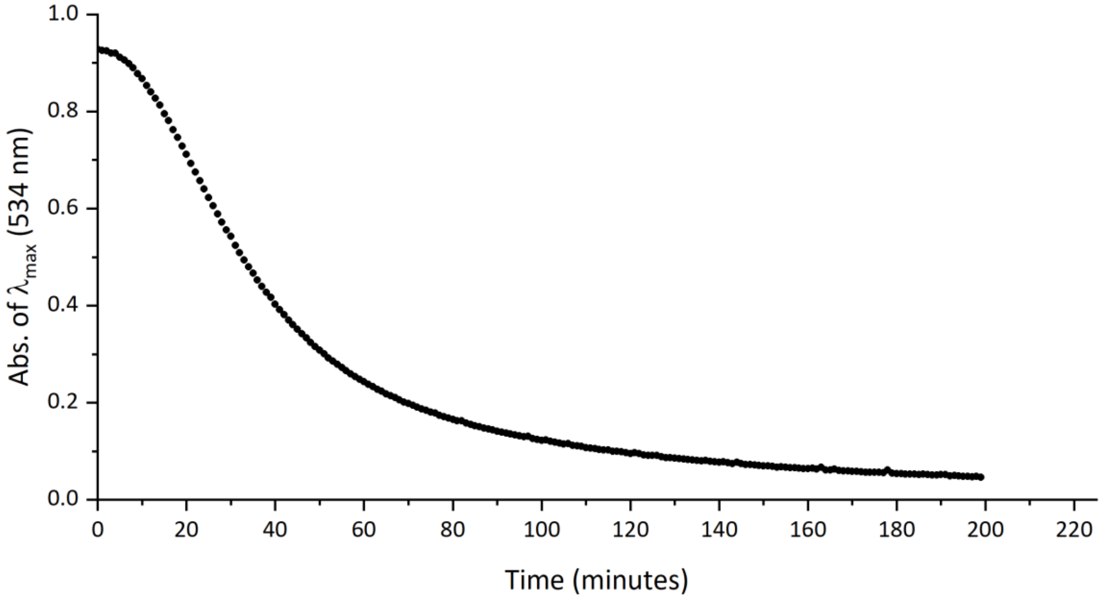

Fig. S26: Change in λ_{max} (534 nm) absorbance of a 10:1 P(mes)$_3$/B(C_6F_5)$_3$ FLP sample (5 mM in toluene) over time under a flow of 5% H_2 & 95% N_2 gas set at 0.1 bar. Half-life ($t_{½}$) = 35 minutes.

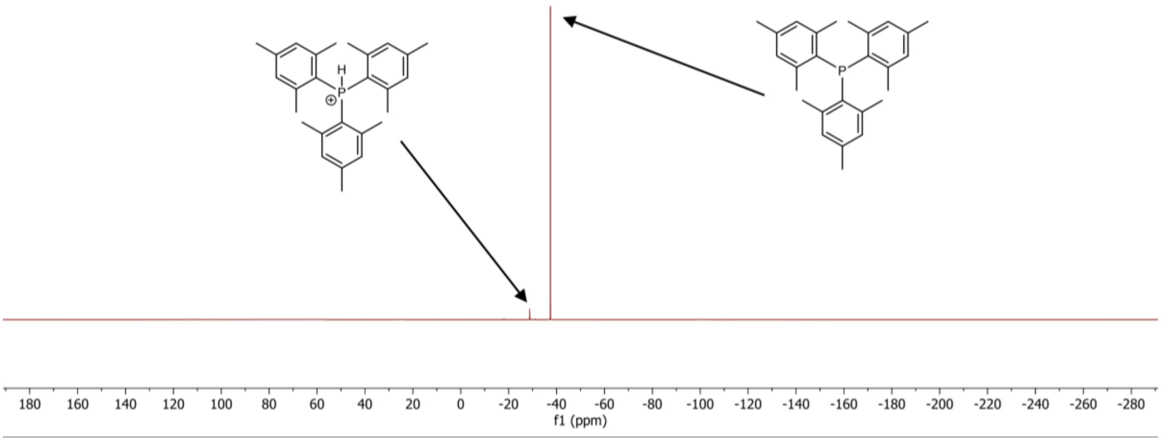

Fig. S27: $^{31}P\{^1H\}$ NMR spectrum of 10:1 $P(mes)_3/B(C_6F_5)_3$ after UV-vis experiments (162 MHz, toluene) δ −37.4 ($P(mes)_3$), −28.8 ($[HP(mes)_3]^+$).

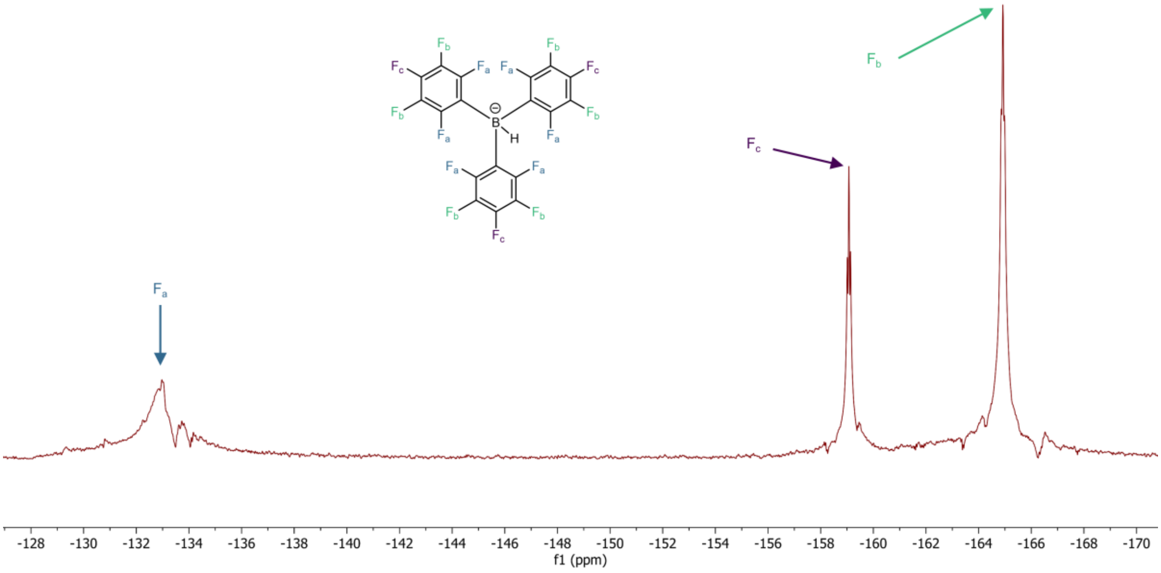

Fig. S28: $^{19}F\{^1H\}$ NMR spectrum of 10:1 $P(mes)_3/B(C_6F_5)_3$ after UV-vis experiments (376 MHz, toluene) δ −133.0 (m, 6F, br, *o*-F), −159.1 (m, 3F, $^3J_{F-F}$ = 22 Hz, *p*-F) −165.0 (m, 6F, $^3J_{F-F}$ = 22 Hz, *m*-F).

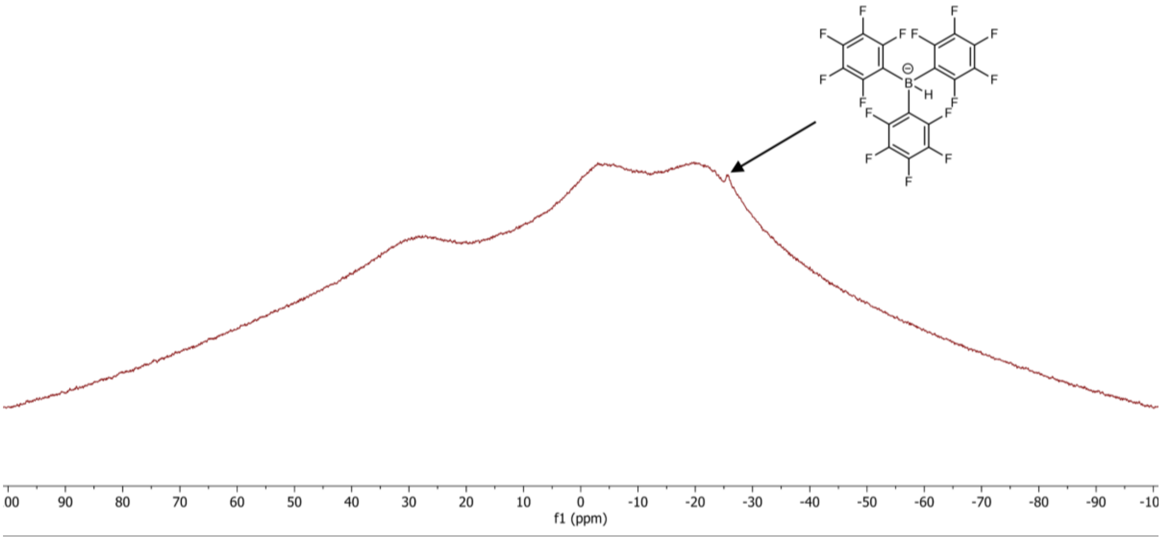

Fig. S29: ^{11}B NMR spectrum of 10:1 $P(mes)_3/B(C_6F_5)_3$ after UV-vis experiments (128 MHz, toluene) δ −25.7 ($[HB(C_6F_5)_3]^-$) (*c.f.* background signal in Fig. S15).

The experiments were repeated for exactly 60 minutes before the ampoule was switched to vacuum to evacuate any residual H_2. The toluene was removed *in vacuo*, and both samples were redissolved in 0.7 mL $CDCl_3$. Percentage conversion was calculated against the equivalents of $P(mes)_3$ in the original solution, using $B(C_6F_5)_3$ as the limiting reagent. These experiments were run using a $^{31}P\{^1H\}$ inverse gated quantitative NMR post programme, with an appropriate relaxation delay (D1 = 200 seconds) to enable accurate integrations of the resonances in the ^{31}P NMR spectrum. This resulted in a conversion to $[HP(mes)_3]^+$ of 25.0% conversion for the 1:1 ($P(mes)_3/B(C_6F_5)_3$) and 41.0% for the 10:1 ($P(mes)_3/B(C_6F_5)_3$) relative to the moles of $B(C_6F_5)_3$ in solution. Conversion rates were also explored by ^1H NMR using 1,3,5-tribromobenzene as an internal standard. Using integration ratios of the internal standard and the $[HP(mes)_3]^+$ signal gave similar results to the ^{31}P QNMR experiment at 29.6% for the 1:1, and 39.3% for the 10:1 respectively ($P(mes)_3/B(C_6F_5)_3$). The difference in values is determined to result from the difficulties in integrating the peaks due to an overlap between the 1,3,5-tribromobenzene peak with one of the doublet peaks of the $[HP(mes)_3]^+$ signal.

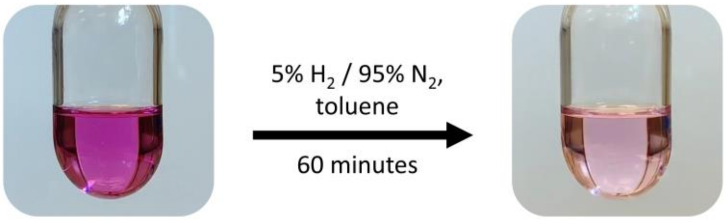

Fig. S30: Visual change in colour of 10:1 $P(mes)_3/B(C_6F_5)_3$ after 60 minutes under a flow of 5% H_2 & 95% N_2 gas.

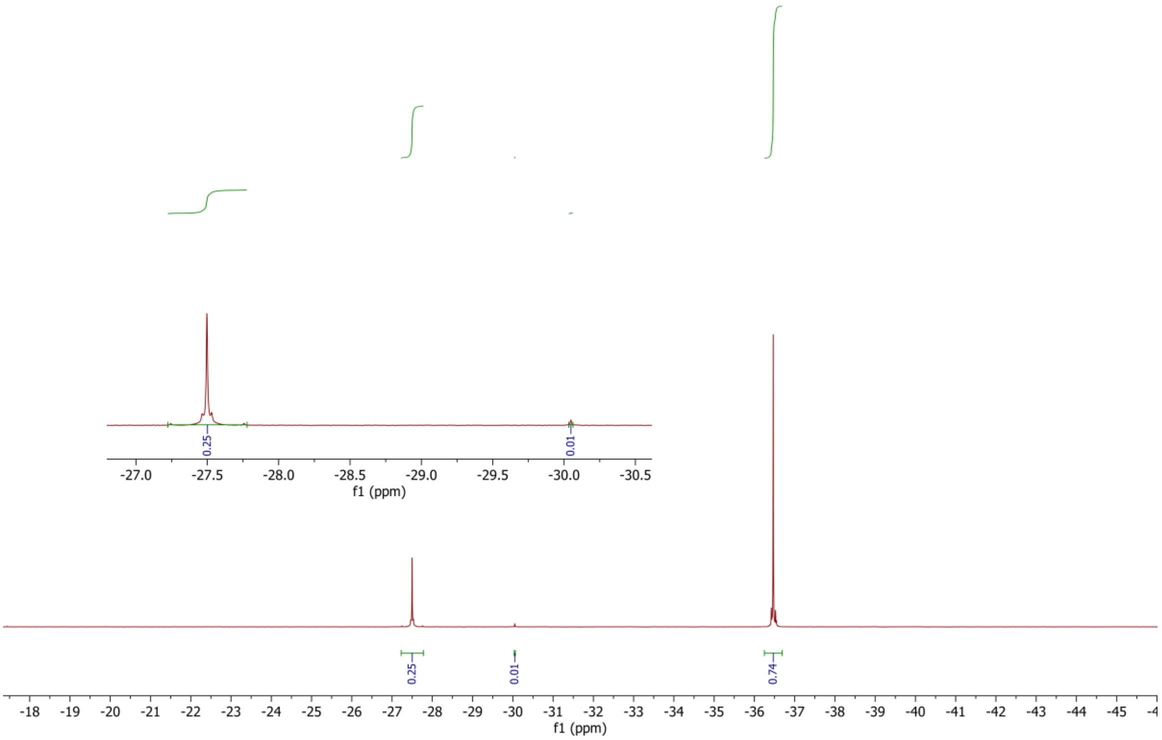

Fig. S31: Quantitative ^{31}P NMR spectrum of 1:1 $P(mes)_3/B(C_6F_5)_3$ after 60 minutes of 5% H_2 & 95% N_2 gas (162 MHz, 298 K, 256 scans, inverse gated, D1 = 200 seconds, $CDCl_3$) δ −36.5 ($P(mes)_3$), −27.5 (d, $[HP(mes)_3]^+$, 25% relative to both $P(mes)_3$ and $B(C_5F_5)_3$). A very minor (1%) contaminant of P_2Mes_4 was observed.

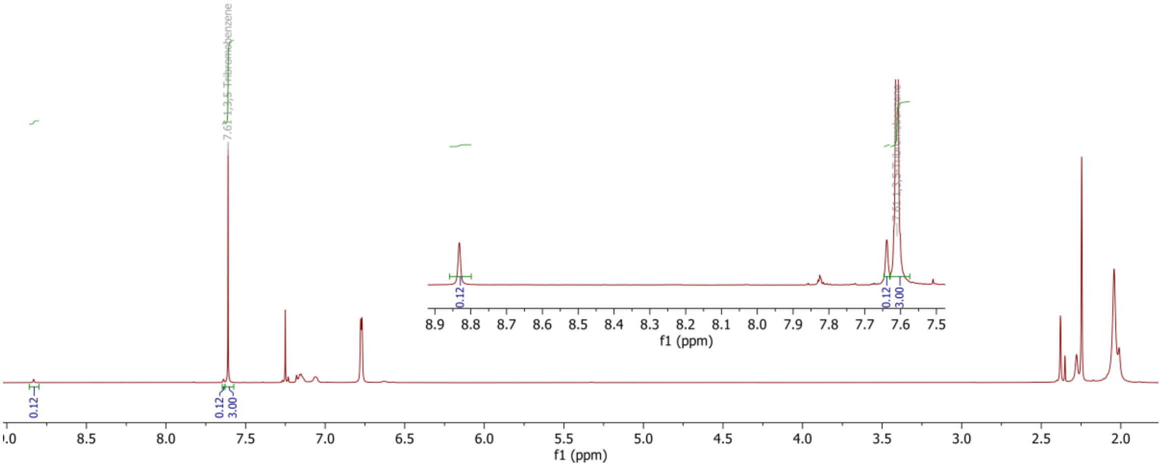

Fig. S32: ^1H NMR spectrum of 1:1 $P(mes)_3/B(C_6F_5)_3$ after 60 minutes of 5% H_2 & 95% N_2 gas (400 MHz, 298 K, 64 scans, D1 = 60 seconds, $CDCl_3$) δ 2.06 (s, 18H, *m*-CH_3), 2.25 (s, 9H, *p*-CH_3), 6.79 (d, $^4J_{H–P}$ = 3.4 Hz, 2H, aryl H), 7.61 (s, 3H, internal standard), 8.26 (d, $^1J_{H–P}$ = 478 Hz, $[HP(mes)_3]^+$, 29.6% relative to internal standard).

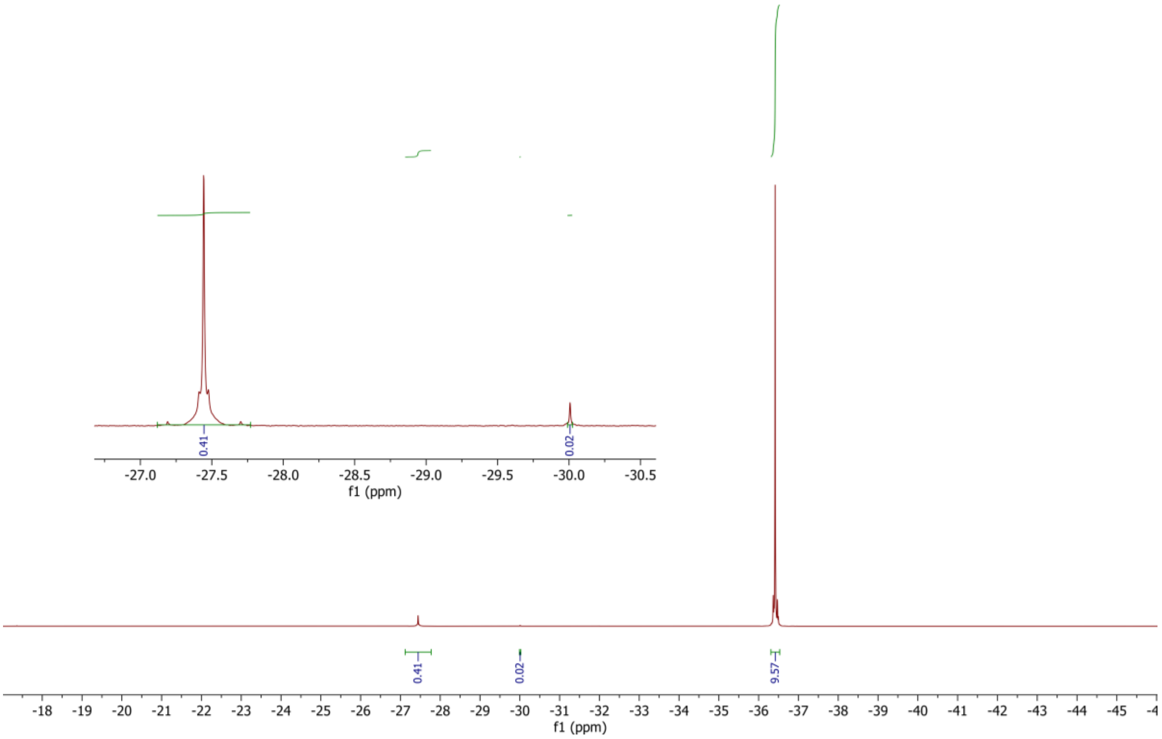

Fig. S33: Quantitative ^{31}P NMR spectrum of 10:1 P(mes)$_3$/B(C_6F_5)$_3$ after 60 minutes of 5% H_2 & 95% N_2 gas (162 MHz, 298 K, 256 scans, inverse gated, D1 = 200 seconds, $CDCl_3$) δ −36.4 (P(mes)$_3$), −27.4 (d, [HP(mes)$_3$]$^+$, 4% relative to P(mes)$_3$, 41% relative to B(C_6F_5)$_3$). A very minor (2%) contaminant of P_2Mes_4 was observed.

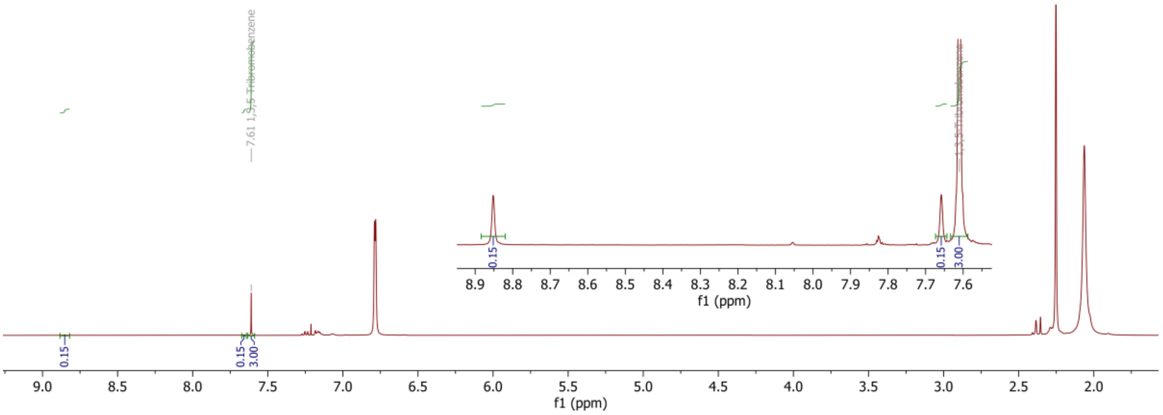

Fig. S34: ^1H NMR spectrum of 10:1 P(mes)$_3$/B(C_6F_5)$_3$ after 60 minutes of 5% H_2 & 95% N_2 gas (400 MHz, 298 K, 64 scans, D1 = 60 seconds, $CDCl_3$) δ 2.05 (s, 18H, *m*-CH_3), 2.25 (s, 9H, *p*-CH_3), 6.79 (d, $^4J_{H\text{–}P}$ = 3.3 Hz, 2H, aryl H), 7.61 (s, 3H, internal standard), 8.24 (d, $^1J_{H\text{–}P}$ = 478 Hz, [HP(mes)$_3$]$^+$, 39.3% relative to internal standard).

Degradation Products

The $P(mes)_3/B(C_6F_5)_3$ FLP is known to be air-sensitive. The magenta colour was lost on exposure to air, and a yellow oil was formed at the bottom of the reaction vessel. NMR spectroscopy was used to probe the degradation products for this FLP combination.

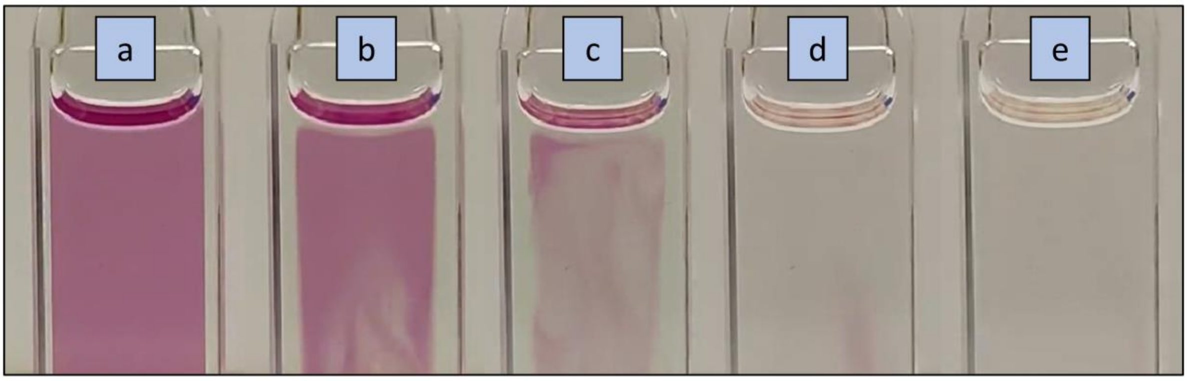

Fig. S35: Photographs from a timelapse of the $P(mes)_3/B(C_6F_5)_3$ complex in toluene degrading in air, a = 0 minutes, b = 5 minutes, c = 8 minutes, d = 11 minutes, e = 13 minutes.

A $P(mes)_3/B(C_6F_5)_3$ sample was exposed to air and left for 48 hours to ensure complete loss of the magenta colour resultant from FLP formation. A pale straw-coloured oil layer formed inside the NMR tube. The remaining toluene was removed *in vacuo* on the Schlenk line, and the oil was re-dissolved in $CDCl_3$ for NMR spectroscopic analysis. The major decomposition pathway is activation of water to afford $[HP(mes)_3][(OH)B(C_6F_5)_3]$,[9, 10, 11] although there are a number of other decomposition product including oxidation of phosphine to give $O{=}P(mes)_3$.[12]

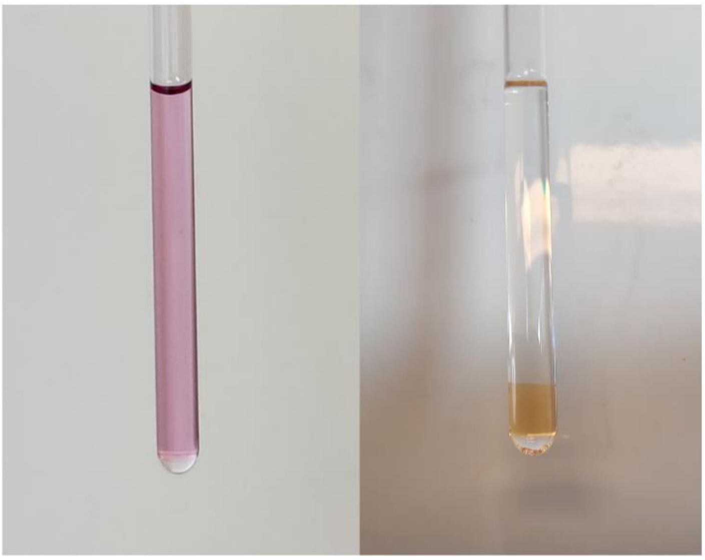

Fig. S36: $P(mes)_3/B(C_6F_5)_3$ sample in toluene before (left), and after (right) when exposed to air (J. Young NMR tube) for 48 hours. Toluene was removed *in vacuo* and remaining oil layer was re-dissolved in $CDCl_3$.

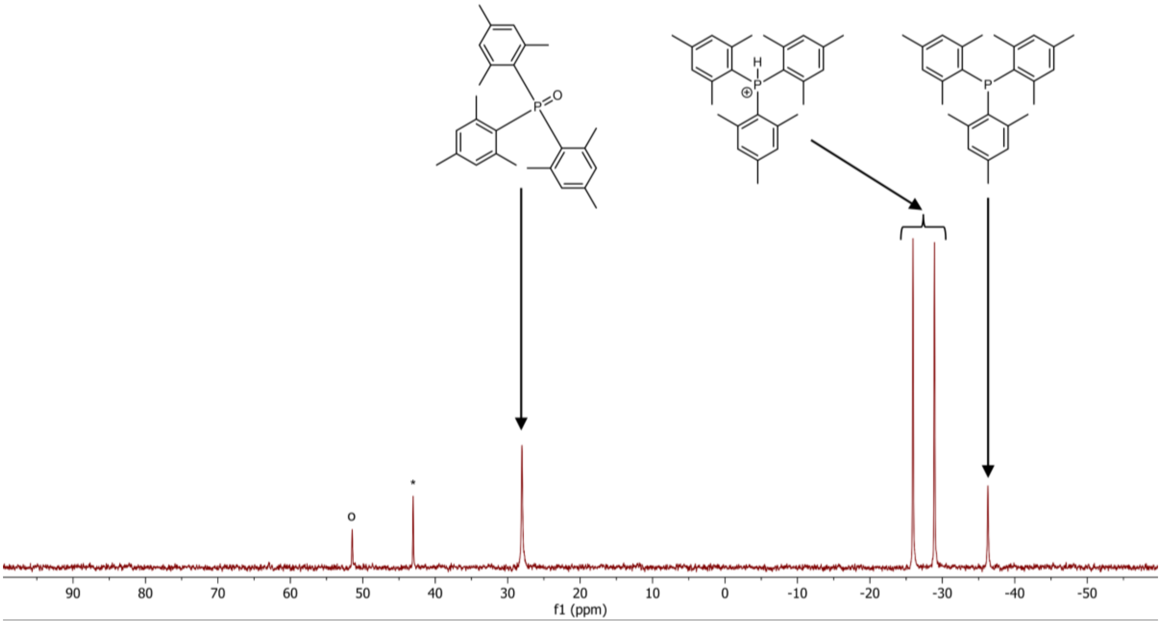

Fig. S37: ^{31}P NMR spectrum of isolated oil layer upon exposure to air (162 MHz, $CDCl_3$) δ −36.3 ($P(mes)_3$), −27.4 (d, $^{1}J_{P\text{-}H}$ = 479 Hz) ($[HP(mes)_3]^+$), 28.0 ($OP(mes)_3$). Unidentified: −43.1 (*), −51.5 (o).

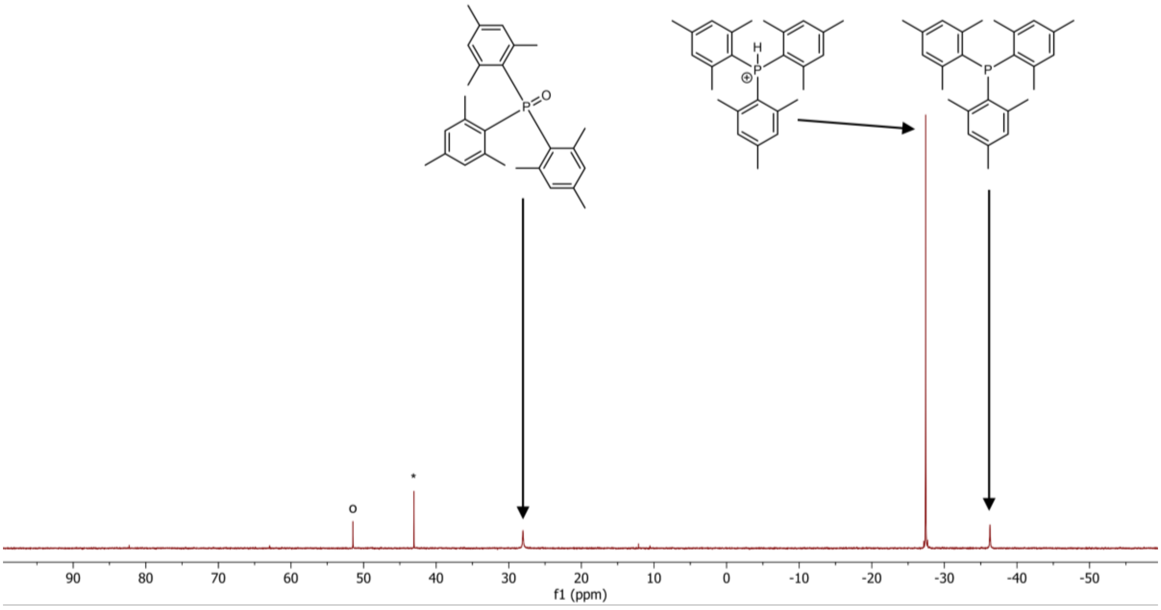

Fig. S38: $^{31}P\{^{1}H\}$ NMR spectrum of isolated oil layer upon exposure to air (162 MHz, $CDCl_3$) δ −36.3 ($P(mes)_3$), −27.4 ($[HP(mes)_3]^+$), 28.0 ($OP(mes)_3$). Unidentified: −43.1 (*), −51.5 (o).

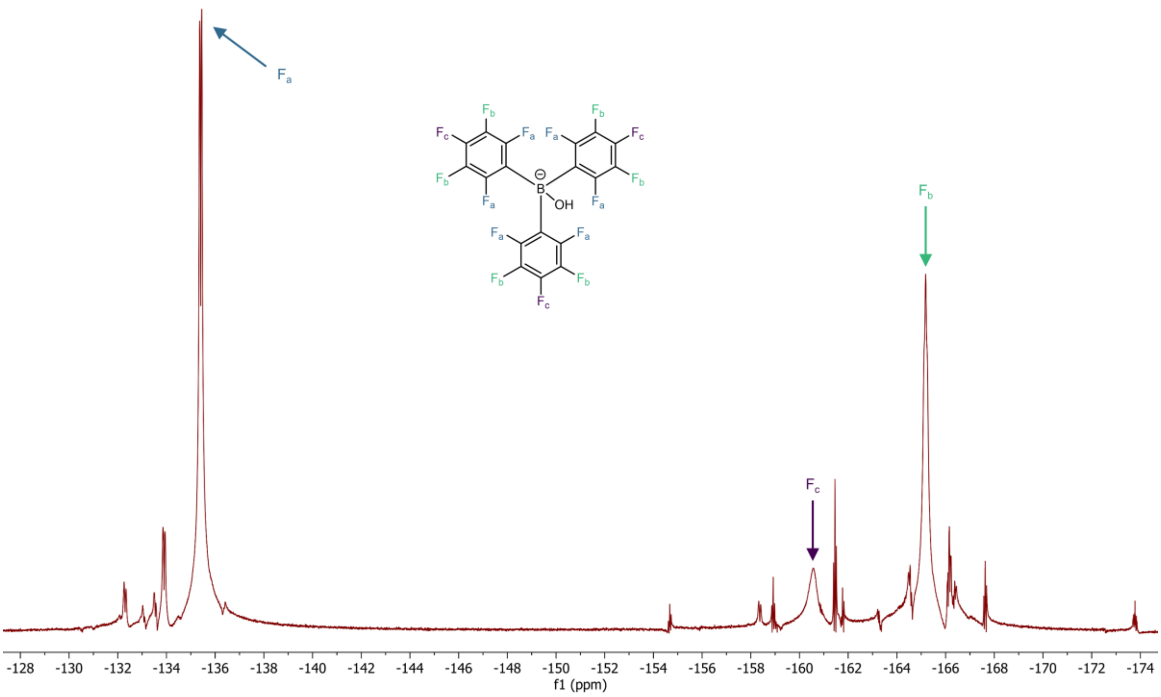

Fig. S39: ^{19}F NMR spectrum of isolated oil layer upon exposure to air (376 MHz, $CDCl_3$) δ −135.4, −160.6, −165.2. There are also a large number of other unidentified decomposition products.

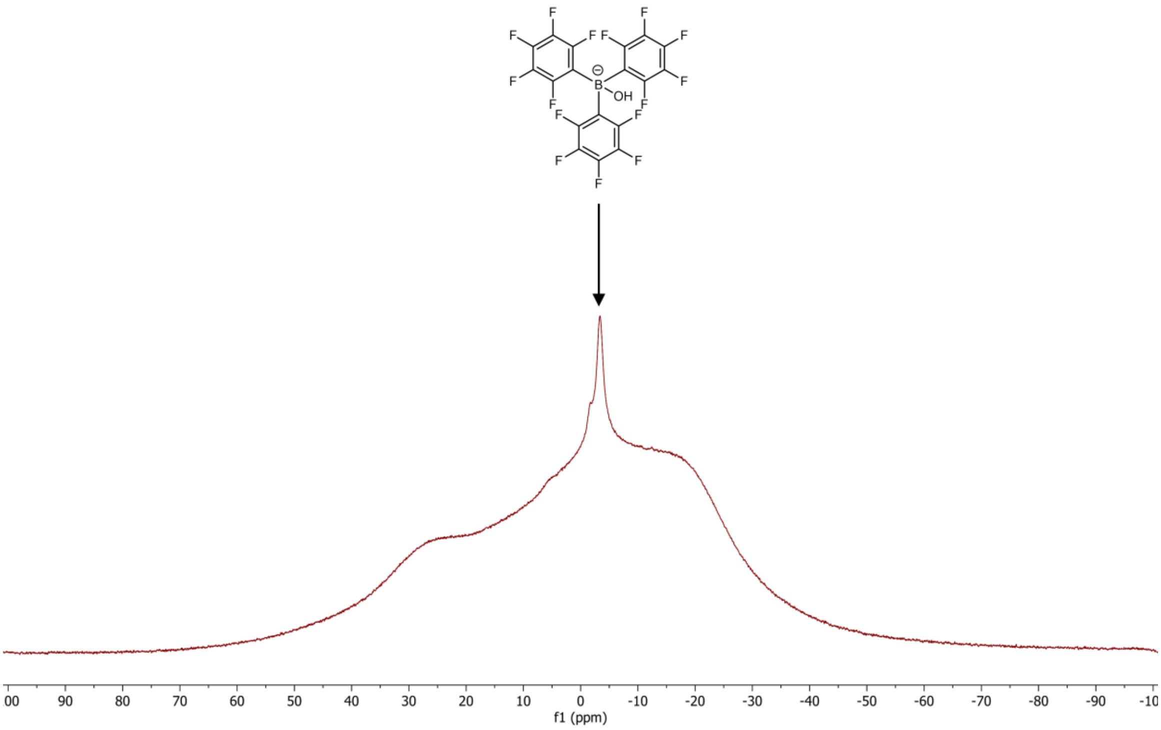

Fig. S40: ^{11}B NMR spectrum of isolated oil layer upon exposure to air (128 MHz, $CDCl_3$) δ −3.4 (*c.f.* background signal in Fig. S15).

Computational Details

Binding configuration generation

Binding configurations were extensively sampled for the association of a frustrated Lewis pair, $P(mes)_3/B(C_6F_5)_3$ (Mes = 2,4,6–$Me_3C_6H_2$), following the procedure described below. Two sampling approaches were used to generate $P(mes)_3/B(C_6F_5)_3$ binding configurations:

(a) Grid sampling

One $B(C_6F_5)_3$ molecule was held stationary, with a regular grid (3 Å grid spacing) created around it. The centre of mass of one $P(mes)_3$ molecule was placed at each of the grid points that were within 18 Å from the centre of mass of the $B(C_6F_5)_3$ molecule. When placed at a grid point, the $P(mes)_3$ molecule was randomly oriented with respect to the $B(C_6F_5)_3$ molecule; eight random orientations of $P(mes)_3$ were sampled at each grid. All configurations in which the $B(C_6F_5)_3$ molecule and the $P(mes)_3$ molecule clashed were discarded.

This procedure was repeated for when the $P(mes)_3$ molecule was held stationary, with the $B(C_6F_5)_3$ molecule sampled around the $P(mes)_3$ molecule. Figure S41 gives a depiction of the grid points around the $P(mes)_3$ molecule that were sampled at for different orientations of the $B(C_6F_5)_3$ molecule.

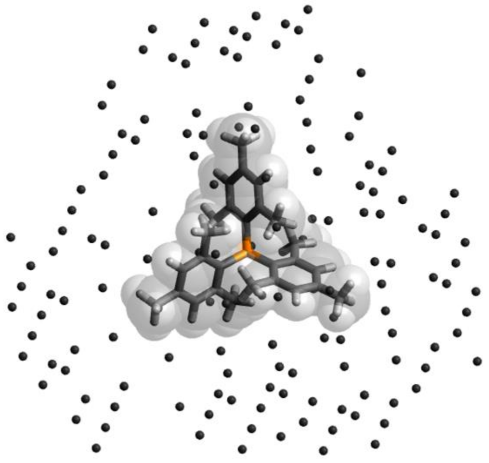

Fig. S41: A depiction of the grid points around the $P(mes)_3$ molecule. The $B(C_6F_5)_3$ molecule was placed at each grid point with eight different random orientations.

A total of 1287 $P(mes)_3/B(C_6F_5)_3$ binding configurations were generated in this grid-sampling approach and fully geometry-optimised using the GFN2-xTB method.[13]

(b) Scanning from known encounter complex configurations

Previously, Grimme and co-workers computationally studied two binding configurations of the $P(mes)_3/B(C_6F_5)_3$ pair,[14] which are referred to as the known encounter complex configurations herein. Figure S42 shows one of the two binding configurations, while the

other one has the $P(mes)_3$ molecule flipped upside-down with the P atom pointing away from the $B(C_6F_5)_3$ molecule.

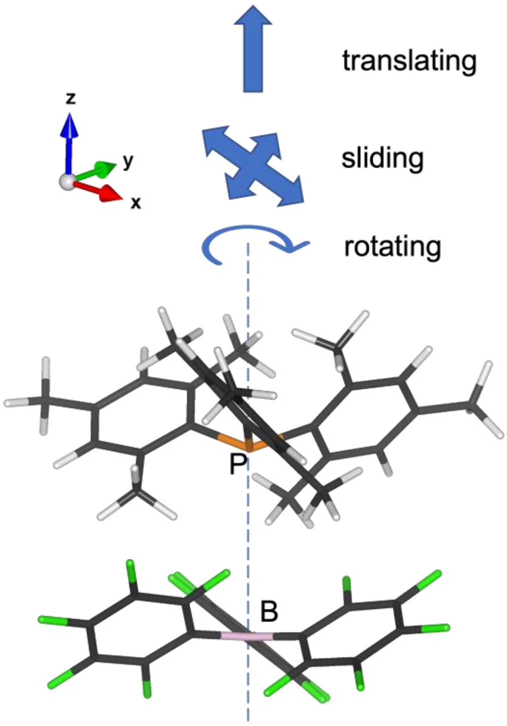

Fig. S42: One encounter complex configuration of the $P(mes)_3/B(C_6F_5)_3$ pair. The arrows indicate the three operations applied to scan binding configurations, starting from the one depicted.

Starting from each of these two known encounter complex configurations, further binding configurations were sampled for the $P(mes)_3/B(C_6F_5)_3$ pair, by applying the following operations to the $B(C_6F_5)_3$ molecule and the $P(mes)_3$ molecule: (1) away from each other along the B–P axial direction (by 0, 3, 6, and 9 Å), (2) rotated around the B–P axial direction (by 0, 30, 60, 90, and 120°), and (3) offset in planes perpendicular to the B–P axis (by 1, 3, and 5 Å in the x, y, −x, and −y directions). After removing configurations in which atoms clashed, a total of 357 binding configurations were scanned, starting from the two known encounter complex configurations, and went through successful geometry optimizations using the same setup as above.

$P(mes)_3/B(C_6F_5)_3$ binding energy landscape

Figure S43 shows the energy landscape of the 1644 $P(mes)_3/B(C_6F_5)_3$ binding configurations (1287 by grid sampling, 357 by targeted scanning), as a function of the distance between the B atom and the P atom of $B(C_6F_5)_3$ and $P(mes)_3$, respectively. All binding energies were calculated using GFN2-xTB, on geometries optimised with GFN2-xTB as implemented by the xTB software.[15] The GFN2-xTB method was used for an initial fast geometry screening and binding energy calculations. Amongst all the sampled binding configurations, the encounter complex configuration shown in Fig. S42 gave the shortest B···P distance.

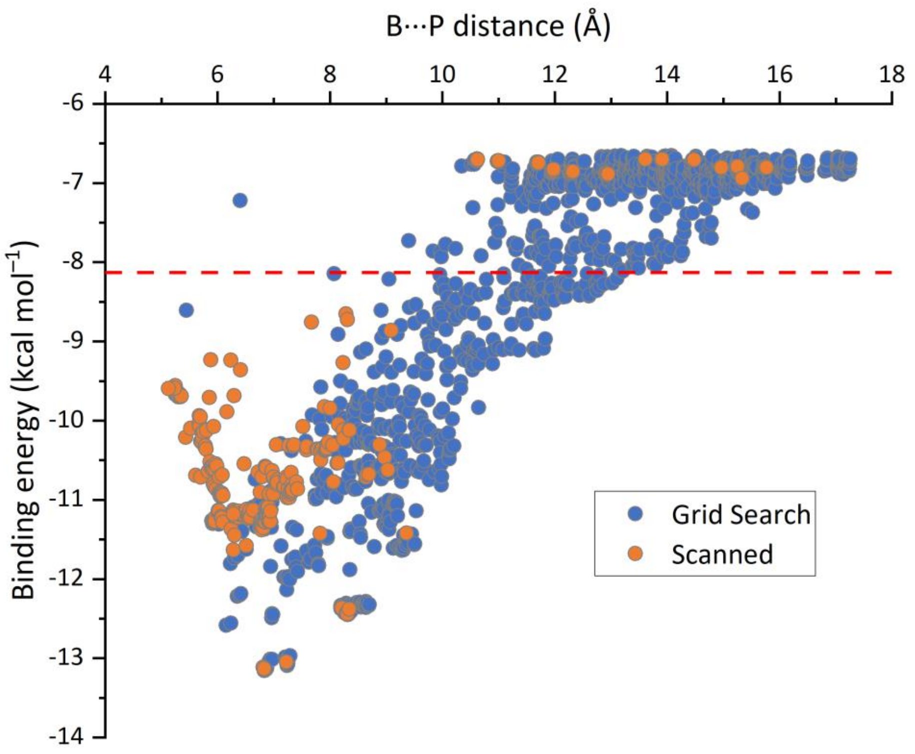

Fig. S43: Binding energy landscape of the $P(mes)_3/B(C_6F_5)_3$ pair, sampled by grid searches (blue dots) and targeted scans (orange dots), at the GFN2-xTB level of theory. The red line represents the cut-off of within 5 kcal mol^{-1} above the global energy minimum.

$P(mes)_3/B(C_6F_5)_3$ binding energy landscape at the density functional theory (DFT) level

By applying a cut-off of within 5 kcal mol^{-1} above the global energy minimum on the xTB binding energy landscape (Fig. S43), 810 xTB-optimised $P(mes)_3/B(C_6F_5)_3$ binding configurations were selected and further geometry-optimised at the B97D3(BJ)/Def2SVP level of theory,[16, 17, 18] with implicit solvation in toluene considered using the PCM/SMD model.[19] B97D3(BJ)/Def2SVP was used for geometry optimisation of 810 structures as the initial step of DFT calculations because it can capture the dispersion interaction with Grimme's D3BJ dispersion at a low cost comparing with ωB97XD used in the next binding energy calculation. 774 of the 810 starting binding configurations were successfully geometry-optimised at the DFT level. The 774 DFT-optimised configurations converged to 72 clusters; structures that converged to the same cluster are exactly, or almost exactly, the same upon visual inspection.

Finally, binding energies of the 774 B97D3(BJ)/Def2SVP-optimised configurations were determined by single-point calculations at the ωB97XD/Def2TZVP level of theory.[17, 18, 20] ωB97XD method and a larger basis set Def2TZVP were used because of accuracy. ωB97XD uses a higher percentage of exact exchange to improve accuracy for short range, and also incorporates 100% Hartree-Fock exchange to better describe long-range effects for the long range. The thus-obtained DFT-based binding energy landscape for the $P(mes)_3/B(C_6F_5)_3$ pair is shown in Fig. S44. The two known encounter complex

configurations were calculated to have binding energies of −10.285 and −10.287 kcal mol^{-1}, respectively, in excellent agreement with the work by Grimme and co-workers[14] (in which they were referred to as 1a and 1b). All calculations were performed using Gaussian 16.[21]

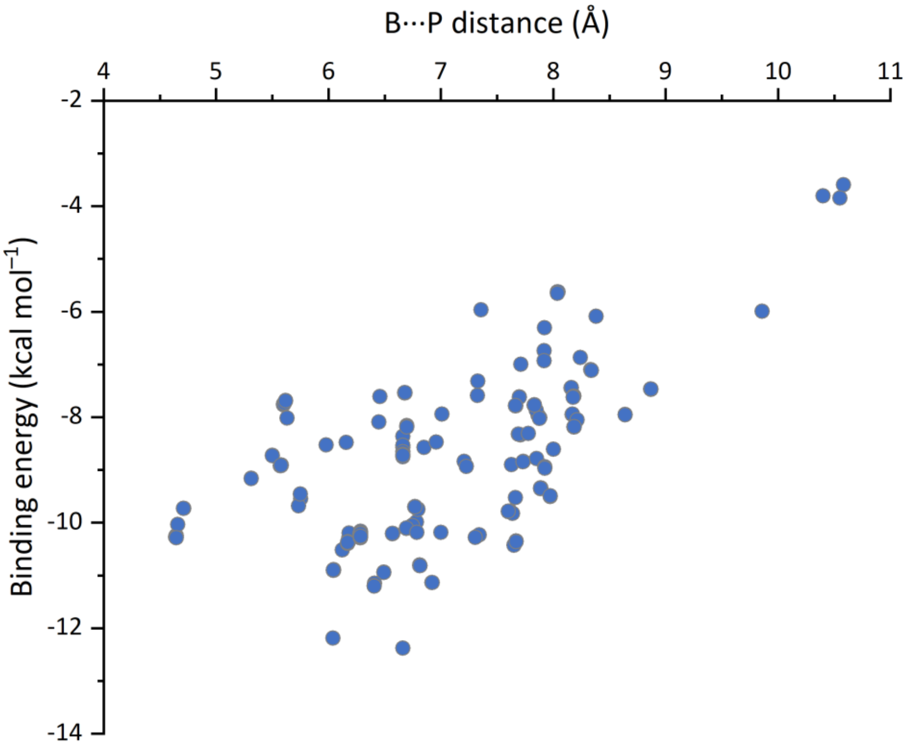

Fig. S44: ωB97XD/Def2TZVP binding energy landscape of the $P(mes)_3/B(C_6F_5)_3$ pair, comprising 774 B97D3(BJ)/Def2SVP-optimised binding configurations (data points representing all 774 configurations are shown in the figure). The 774 xTB-optimised, starting geometries converged to 72 clusters of geometries.

Time-dependent DFT (TD-DFT) calculations

The 774 B97D3(BJ)/Def2SVP-optimised binding configurations of the $P(mes)_3/B(C_6F_5)_3$ pair converged to 72 clusters of binding configurations (Fig. S44). The lowest energy configuration of each cluster was selected to form a set of 72 representative binding configurations. Vertical excitation was then simulated for each of the 72 representative binding configurations, at the M06-2X/Def2SVP level of theory.[17, 18, 22] The effect of solvation by toluene was accounted for by using the PCM/SMD solvation model. M06-2X is good for the charge separation calculations because of its higher HF exchange comparing to M06 (below) and B97XD (above), so it is accurate in the TDDFT calculations related to electronic excitation energies.

Figure S44a reports the excitation energy of the first excited state (S_1) for the 72 representative $P(mes)_3/B(C_6F_5)_3$ binding configurations, as a function of the B···P distance. The S_1 absorption wavelength decreases—*i.e.*, S_1 excitation energy increases—nearly monotonically with increasing separation distance between the B atom and the P atom of $B(C_6F_5)_3$ and $P(mes)_3$, respectively. The encounter complex configuration shown in Fig. S42 was calculated to exhibit the lowest S_1 excitation energy (499 nm) within the set (Molecular ID: 804). It is worth noting, there are 4 other structures that display a 'slipped'

configuration from the structure shown in Fig. S42, Molecular IDs: 627, 629 (1' in Fig. S45b, see discussion below), 641, and 655—all of which are relatively minor deviations from the active encounter complex and could feasibly activate H_2. These four structures have S_1 excitation energies of 478, 493, 479, and 478 nm respectively, which could be contributing to the broad CT-band observed for the encounter complex, while remaining catalytically relevant. For the encounter complex (Molecular ID: 804), natural transition orbitals (NTOs) were generated and are shown in Fig. S47. There is an almost exclusive (99.86%) NTO transition from $P(mes)_3$ to $B(C_6F_5)_3$, showing an almost complete charge redistribution between the two molecules upon electronic excitation (Fig. S47).

In addition to these 72 data points, another data point was added that was from the same cluster as the “active encounter complex”. The original data point from that cluster is labelled as 1 in Fig. S45, and the additional data point is labelled 1'. It is clear from the structures in Fig. S45b that these are visually very similar, and these small changes have minor effects on the calculated properties.

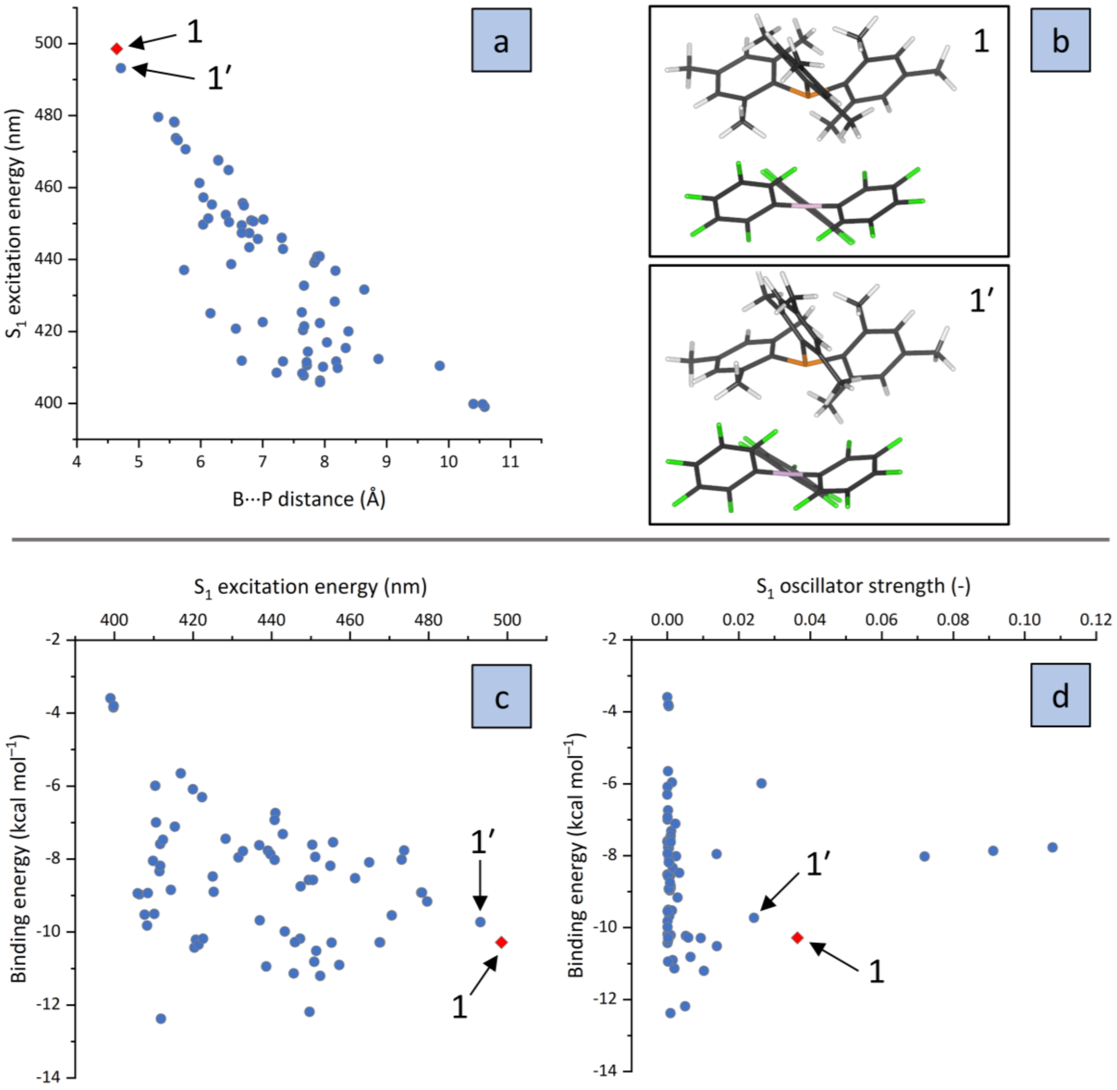

Fig. S45: (a) First excited state (S_1) excitation energy as a function of the B⋯P distance for the 72 representative binding configurations of the $P(mes)_3/B(C_6F_5)_3$ pair. (b) Binding configurations corresponding to the data points that are labelled 1 and 1′ in (a), (c), and (d). (c) Binding energy plotted against S_1 excitation energy. (d) Binding energy plotted against S_1 oscillator strength. The red diamond represents the “active encounter complex”.

By applying a cut-off at −10 kcal mol^{-1} in Fig. S44, 13 P(mes)$_3$/B(C_6F_5)$_3$ binding configurations were selected and further investigated using the M06/6-311G(2df,p) level of theory with the D3 version of Grimme's dispersion correction,[22, 23, 24, 25] permitting the capture of the relevant entropic effects at T=301K, corresponding to the experimental measurement temperature. Gibbs free energies calculations are time consuming, so M06 method was used to calculate Gibbs free energies of these points at an acceptable time scale. M06 has lower HF exchange compared to M06-2X, which makes the calculation faster.

ID	ΔE (kcal mol^{-1})	ΔG (kcal mol^{-1})
709	-18.3314817	2.5990083
300	-23.8260771	-0.3239271
273	-22.8952773	-1.0745973
231	-20.8895022	0.4070178
525	-21.2494401	-0.7423101
504	-20.806632	-0.392112
122	-20.266659	0.4638501
479	-22.1661972	-0.8199072
141	-20.0470914	0.7347186
246	-20.2245561	1.9942839
338	-20.9056806	1.6149294
776	-21.4604208	0.3234483
804	-18.9157374	-2.8790874

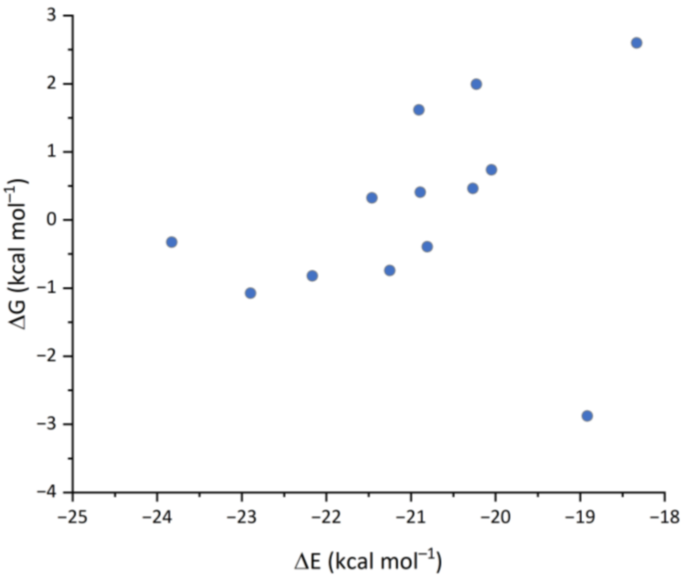

Fig. S46: Tabulated and graphical comparison of ΔE and ΔG of the P(mes)$_3$/B(C_6F_5)$_3$ pair calculated by M06/6-311G(2df,p) with D3 version of Grimme's dispersion. Molecular ID 804 is the 'face toward' orientation (Fig S45 b1).

Additional Figures

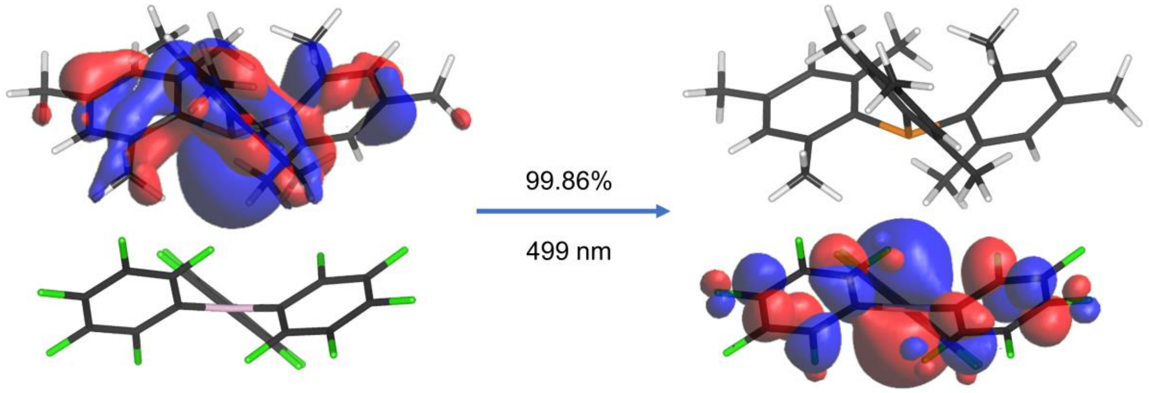

Fig. S47: Natural transition orbitals (NTOs) for the dominant component transition of the first excited state of the $P(mes)_3/B(C_6F_5)_3$ encounter complex configuration shown in Fig. S42, with the corresponding contribution (99.86%) from the NTO pair labelled above the arrow indicating the transition.

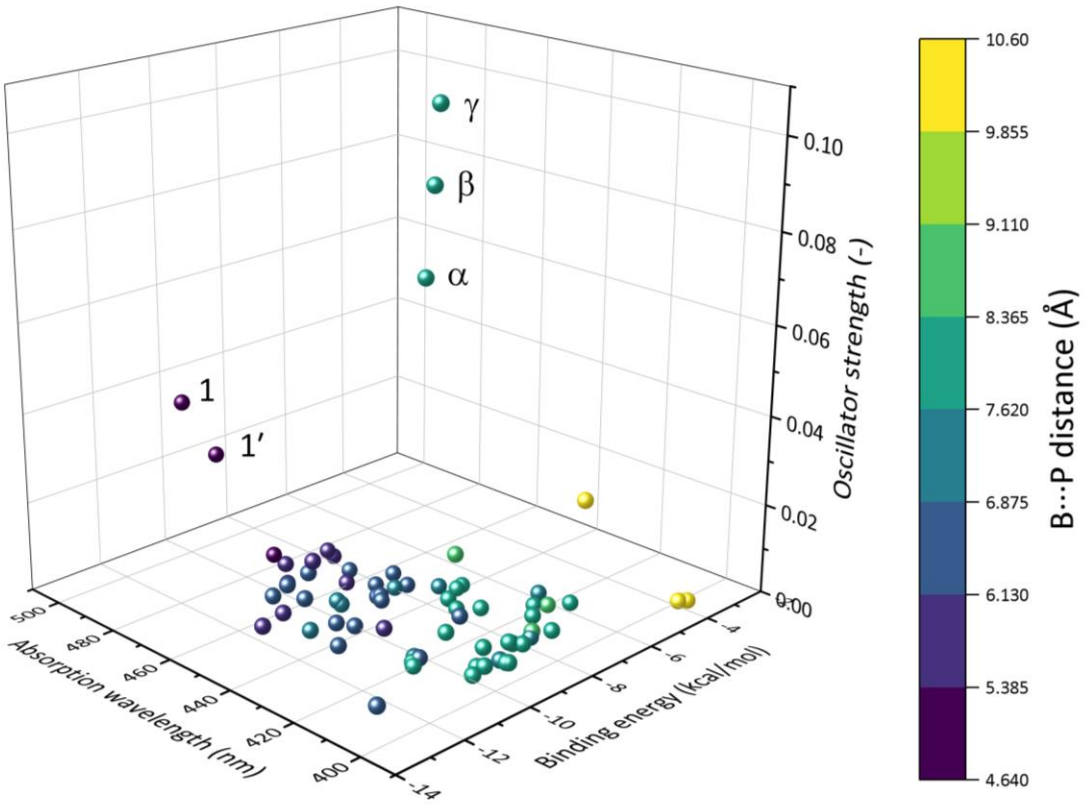

Fig. S48: 3D representation of Binding energy (kcal mol^{-1}) vs. S_1 excitation energy and oscillator strength for $P(mes)_3/B(C_6F_5)_3$. The colour of the datapoints corresponds to the B···P distance (Å). 1 is the "active encounter complex" form, with 1′ being a slight variation taken from the same cluster (Fig. S45b). Datapoints α, β, and γ correspond to three π-stacking conformations (Molecular IDs: 529, 466, and 349 respectively) (Fig. S49).

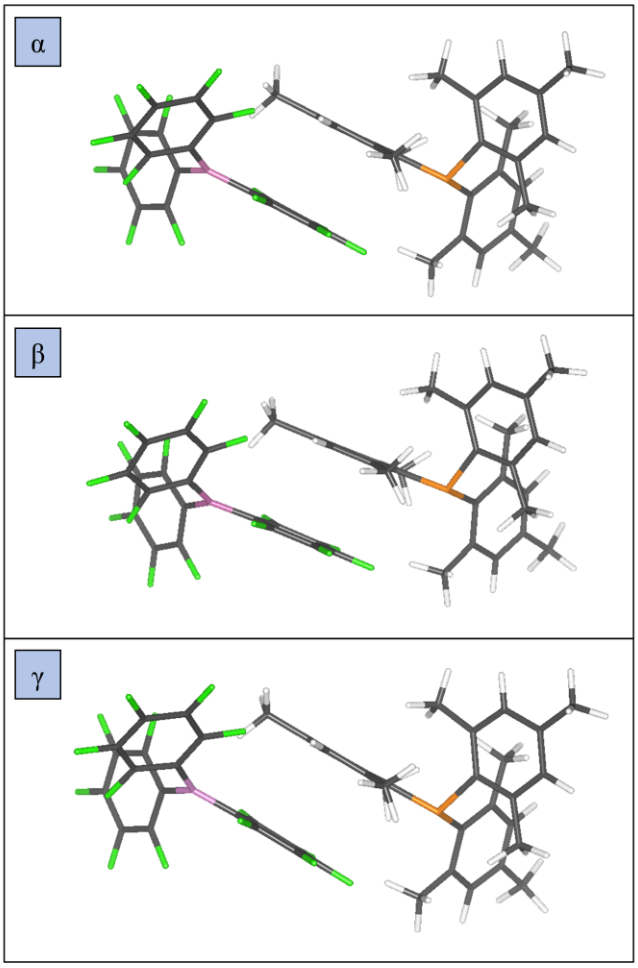

Fig. S49: 3D representation of α (ID: 529), β (ID: 466), and γ (ID: 349) ($P(mes)_3/B(C_6F_5)_3$) showing π-stacking interactions. All configurations are geometrically similar with only small differences in the B–P distance.

References

1. Trunk M, Teichert JF, Thomas A. Room-Temperature Activation of Hydrogen by Semi-immobilized Frustrated Lewis Pairs in Microporous Polymer Networks. *J. Am. Chem. Soc.* 2017, **139**(10)**:** 3615-3618.

2. Stepanov BI, Bokanov AI, Kudryavtsev AB, Plyashkevich YG. Organophosphorus Derivatives of Durele. *Zh. Obshch. Khim.* 1974, **44:** 2358.

3. Negrebetskii VV, Bogel'fer LY, Bokanov AI, Rozanel'skaya NA, Stepanov BI. Influence of the stereoisomerization of triarylphosphines on the parameters of the ^{13}C NMR spectra. *J. Struct. Chem.* 1979, **19**(4)**:** 545-548.

4. Reißmann M, Schäfer A, Jung S, Müller T. Silylium Ion/Phosphane Lewis Pairs. *Organometallics* 2013, **32**(22)**:** 6736-6744.

5. Soltani Y, Wilkins LC, Melen RL. Stoichiometric and Catalytic C−C and C−H Bond Formation with $B(C_6F_5)_3$ via Cationic Intermediates. *Angew. Chem. Int. Ed.* 2017, **56**(39)**:** 11995-11999.

6. www.supramolecular.org.

7. Brynn Hibbert D, Thordarson P. The death of the Job plot, transparency, open science and online tools, uncertainty estimation methods and other developments in supramolecular chemistry data analysis. *Chem. Commun.* 2016, **52**(87)**:** 12792-12805.

8. Ulatowski F, Dąbrowa K, Bałakier T, Jurczak J. Recognizing the Limited Applicability of Job Plots in Studying Host–Guest Interactions in Supramolecular Chemistry. *J. Org. Chem.* 2016, **81**(5)**:** 1746-1756.

9. Welch GC, Stephan DW. Facile Heterolytic Cleavage of Dihydrogen by Phosphines and Boranes. *J. Am. Chem. Soc.* 2007, **129**(7)**:** 1880-1881.

10. Jiang C, Blacque O, Berke H. Metal-Free Hydrogen Activation by the Frustrated Lewis Pairs of $ClB(C_6F_5)_2$ and $HB(C_6F_5)_2$ and Bulky Lewis Bases. *Organometallics* 2009, **28**(17)**:** 5233-5239.

11. Fasano V, Ingleson MJ. Expanding Water/Base Tolerant Frustrated Lewis Pair Chemistry to Alkylamines Enables Broad Scope Reductive Aminations. *Chem. Eur. J.* 2017, **23**(9)**:** 2217-2224.

12. Laye C, Lusseau J, Robert F, Landais Y. The Trityl-Cation Mediated Phosphine Oxides Reduction. *Adv. Synth. Catal.* 2021, **363**(12)**:** 3035-3043.

13. Bannwarth C, Ehlert S, Grimme S. GFN2-xTB—An Accurate and Broadly Parametrized Self-Consistent Tight-Binding Quantum Chemical Method with Multipole Electrostatics and Density-Dependent Dispersion Contributions. *J. Chem. Theory Comput.* 2019, **15**(3)**:** 1652-1671.

14. Bannwarth C, Hansen A, Grimme S. The Association of Two “Frustrated” Lewis Pairs by State-of-the-Art Quantum Chemical Methods. *Isr. J. Chem.* 2015, **55**(2)**:** 235-242.

15. Bannwarth C, Caldeweyher E, Ehlert S, Hansen A, Pracht P, Seibert J*, et al.* Extended tight-binding quantum chemistry methods. *WIREs Comput. Mol. Sci* 2021, **11**(2).

16. Becke AD. Density-functional thermochemistry. V. Systematic optimization of exchange-correlation functionals. *J. Chem. Phys.* 1997, **107**(20)**:** 8554-8560.

17. Weigend F, Ahlrichs R. Balanced basis sets of split valence, triple zeta valence and quadruple zeta valence quality for H to Rn: Design and assessment of accuracy. *Phys. Chem. Chem. Phys.* 2005, **7**(18)**:** 3297-3305.

18. Weigend F. Accurate Coulomb-fitting basis sets for H to Rn. *Phys. Chem. Chem. Phys.* 2006, **8**(9)**:** 1057.

19. Marenich AV, Cramer CJ, Truhlar DG. Universal Solvation Model Based on Solute Electron Density and on a Continuum Model of the Solvent Defined by the Bulk Dielectric Constant and Atomic Surface Tensions. *J. Phys. Chem. B* 2009, **113**(18)**:** 6378-6396.

20. Chai JD, Head-Gordon M. Long-range corrected hybrid density functionals with damped atom-atom dispersion corrections. *Phys. Chem. Chem. Phys.* 2008, **10**(44)**:** 6615-6620.

21. Frisch MJ, Trucks GW, Schlegel HB, Scuseria GE, Robb MA, Cheeseman JR*, et al.* Gaussian 16 Rev. C.01. Wallingford, CT; 2016.

22. Zhao Y, Truhlar DG. The M06 suite of density functionals for main group thermochemistry, thermochemical kinetics, noncovalent interactions, excited states, and transition elements: two new functionals and systematic testing of four M06-class functionals and 12 other functionals. *Theor. Chem. Acc.* 2007, **120**(1-3)**:** 215-241.

23. Grimme S, Antony J, Ehrlich S, Krieg H. A consistent and accurate *ab initio* parametrization of density functional dispersion correction (DFT-D) for the 94 elements H-Pu. *J. Phys. Chem.* 2010, **132**(15)**:** 154104.

24. McLean AD, Chandler GS. Contracted Gaussian basis sets for molecular calculations. I. Second row atoms, *Z*=11–18. *J. Chem. Phys.* 1980, **72**(10)**:** 5639-5648.

25. Krishnan R, Binkley JS, Seeger R, Pople JA. Self-consistent molecular orbital methods. XX. A basis set for correlated wave functions. *J. Chem. Phys.* 1980, **72**(1)**:** 650-654.
